# Stochastic and Differential Activation of σ^B^ and PrfA in *Listeria monocytogenes* at the Single Cell Level under Different Environmental Stress Conditions

**DOI:** 10.3389/fmicb.2017.00348

**Published:** 2017-03-14

**Authors:** Claudia Guldimann, Veronica Guariglia-Oropeza, Sophia Harrand, David Kent, Kathryn J. Boor, Martin Wiedmann

**Affiliations:** Food Safety Laboratory, Department of Food Science, Cornell UniversityIthaca, NY, USA

**Keywords:** *Listeria monocytogenes*, PrfA, sigB, single cell, stochastic gene expression, heat stress, salt stress

## Abstract

During host infection, the foodborne pathogen *Listeria monocytogenes* must sense and respond to rapidly changing environmental conditions. Two transcriptional regulators, the alternative sigma factor B (σ^B^) and the Positive Regulatory Factor A (PrfA), are key contributors to the transcriptomic responses that enable bacterial survival in the host gastrointestinal tract and invasion of host duodenal cells. Increases in temperature and osmolarity induce activity of these proteins; such conditions may be encountered in food matrices as well as within the host gastrointestinal tract. Differences in PrfA and σ^B^ activity between individual cells might affect the fate of a cell during host invasion, therefore, we hypothesized that PrfA and σ^B^ activities differ among individual cells under heat and salt stress. We used fluorescent reporter fusions to determine the relative proportions of cells with active σ^B^ or PrfA following exposure to 45°C heat or 4% NaCl. Activities of both PrfA and σ^B^ were induced stochastically, with fluorescence levels ranging from below detection to high among individual cells. The proportion of cells with active PrfA was significantly higher than the proportion with active σ^B^ under all tested conditions; under some conditions, nearly all cells had active PrfA. Our findings further support the growing body of evidence illustrating the stochastic nature of bacterial gene expression under conditions that are relevant for host invasion via food-borne, oral infection.

## Introduction

*Listeria monocytogenes* is a foodborne pathogen responsible for bacterial infections in many species including ruminants and humans. While the incidence of listeriosis is relatively low [0.26 cases/1,000,000 in the USA in 2013 (Crim et al., 2015)], a high fatality rate, estimated to be ~15–30 deaths per 100 cases (de Valk et al., 2005; Popovic et al., 2014; Crim et al., 2015), makes *L. monocytogenes* a major food safety concern in many industrialized countries, particularly because it affects vulnerable populations including pregnant women, infants, and individuals with suppressed immune systems. Infection is typically acquired by ingestion of contaminated food with subsequent invasion of cells in the host duodenum. *L. monocytogenes* can enter the bloodstream and spread systemically, including to the placenta and the central nervous system. Consequently, listeriosis may present as gastroenteritis, septicemia, neonatal infection, or meningoencephalitis and can cause abortion in pregnant individuals (reviewed in Allerberger and Wagner, 2010).

*L. monocytogenes* is able to transition from a saprophytic lifestyle in the environment to an intracellular niche within host cells, which requires adaptation to the rapidly changing conditions encountered along the oral infection route, including shifts in temperature, pH, and osmolarity. These adaptation processes require substantial changes in gene expression. Two transcription factors, the alternative sigma factor B (σ^B^) and the Positive Regulatory Factor A (PrfA), are central nodes in a complex regulatory network that governs the remodeling of the transcriptome during host infection (Chaturongakul et al., 2008; De las Heras et al., 2011). σ^B^ activates transcription of a large regulon that includes stress response as well as some virulence genes (Wiedmann et al., 1998); PrfA is the primary transcriptional regulator of virulence genes (Mengaud et al., 1991). A growing body of research shows that even within a clonal population of bacterial cells exposed to a superimposed environmental condition, bacterial gene expression in a population is inherently noisy, often showing stochastic patterns (reviewed in Kaern et al., 2005; Raj and van Oudenaarden, 2008). In *L. monocytogenes*, stochastic activity of σ^B^ in the presence of 0.5 M salt and in stationary phase has been previously demonstrated (Utratna et al., 2012). Such stochastic differences in gene expression can create specialized subpopulations that may serve the purpose of “labor-sharing,” where only a few cells carry the burden of producing a molecule that will profit the entire population, or “bet-hedging,” where the individual cells bearing the expressed molecules will have a relatively enhanced chance of surviving future abrupt changes in environmental parameters (Eldar and Elowitz, 2010).

A previous study in a pregnant guinea pig model has shown that a very small number of *L. monocytogenes* cells traffic from the maternal organs to the placenta; further, placental infection can originate from a single invading cell (Bakardjiev et al., 2006). Why would one individual cell succeed in invading the host (e.g., during the gastrointestinal stage of infection) while others do not? Previous high-resolution transcriptomics studies documenting the transcriptional changes in *L. monocytogenes* upon host infection (Camejo et al., 2009; Toledo-Arana et al., 2009) do not account for stochastic differences at the single cell level. Information on gene expression patterns in individual bacteria that succeed to establish infection could therefore provide novel insights into host-pathogen interactions at very high resolution. However, for *L. monocytogenes* there is no data on how differential gene expression affects the ability of individual cells to ultimately succeed in host invasion. The aim of this study was to take a first step toward addressing this question. Specifically, we hypothesized that σ^B^ and PrfA activities differ in individual bacteria in response to several environmental conditions. We chose two conditions shown previously to induce σ^B^ activity: heat or salt stress (i.e., exposure to 45°C or 4.4% NaCl) (Sue et al., 2003; Hu et al., 2007; Abram et al., 2008; Somolinos et al., 2010; Ait-Ouazzou et al., 2012; Ringus et al., 2012; Ribeiro et al., 2014). Water phase salt concentrations in the range of 4% are typical in food preservation, and salt is used in the preservation of foods linked to human listeriosis cases and outbreaks (e.g., cheese or deli meat; Gottlieb et al., 2006; Currie et al., 2015; Heiman et al., 2016; McIntyre et al., 2015). Heat stress at 45°C was used as a model for another, non-lethal stress condition. To probe the single cell response to the selected environmental conditions, we used fluorescent reporter fusions to visualize the activities of σ^B^ and PrfA in response to heat and salt stress.

## Materials and methods

### Bacterial strains and plasmids

*E. coli* was grown in Luria Bertani (LB) broth or on LB agar (at 37°C), while *L. monocytogenes* was grown in brain heart infusion (BHI) broth or on BHI agar at 37°C unless otherwise stated. Stock cultures of all strains (see Table 1 for a list of strains) were stored at −80°C in LB or BHI broth with 15% glycerol. From those stocks, cultures were streaked on LB or BHI agar plates to obtain single colonies to inoculate overnight cultures. For stress exposure experiments, cultures were started from frozen stock and grown on BHI agar. After overnight incubation, a single colony was inoculated into 5 ml BHI and grown for 6–8 h. This culture was then diluted 1:1000 into fresh BHI and grown for 15–17 h. This stationary phase culture was then diluted 1:100 into 100 ml BHI and the resulting culture was grown to either OD600 = 0.4 (representing mid-log phase) or OD600 = 1.0 + 1 h (early stationary phase). All cultures were grown with aeration (shaking at 200 rpm). Where appropriate, chloramphenicol (at 25 μg/ml) or kanamycin (at 30 μg/ml) was added to the growth media during the construction of the reporter strains.

**Table 1:** **Bacterial strains**.

**Parental/host strain**	**Species**	**strain**	**Relevant Genotype**
*Listeria monocytogenes*	10403S	wt	Bishop and Hinrichs, 1987
*Listeria monocytogenes*	10403S	Δ*sigB*	Wiedmann et al., 1998
*Listeria monocytogenes*	10403S	Δ*prfA*	Wong and Freitag, 2004
*Listeria monocytogenes*	EGDe	P*lmo2230::eGFP*	Utratna et al., 2012
*Listeria monocytogenes*	10403S	P*hly::eGFP*	this study
*Listeria monocytogenes*	10403S	P*lmo2230::eGFP*	this study
*Listeria monocytogenes*	10403S	constitutive GFP	this study
*Listeria monocytogenes*	10403S	Δ*sigB*, P*hly::eGFP*	this study
*Listeria monocytogenes*	10403S	Δ*prfA*, P*hly::eGFP*	this study
*Listeria monocytogenes*	10403S	Δ*sigB*, P*lmo2230::eGFP*	this study
*E.coli* COB631	Top ten	pKSV-7-*lmo2230::eGFP*	Utratna et al., 2012
*E.coli*	DH5α	pPL2-*hly::eGFP* (pCG8)	this study
*E.coli*	SM10	pPL2-*hly::eGFP* (pCG8)	this study
*E.coli*	DH5α	pPL2-*lmo2230::eGFP* (pCG1)	this study
*E.coli*	SM10	pPL2-*lmo2230::eGF*P (pCG1)	this study

### Cloning techniques and construction of plasmids

Primers and plasmids used in this study are listed in Table 2, Supplementary Table 1. PCRs were carried out using Q5 polymerase (NEB, Ipswich, MA) according to the manufacturer's protocol; when necessary PCR products were purified using Qiagen (Valencia, CA) PCR Purification Kit No. 28106. For gel extractions, Invitrogen (Carlsbad, CA) Kit No. K210012 was used. Restriction digests were carried out using enzymes purchased from NEB. Plasmids were isolated using Qiagen Kit No. 27104.

**Table 2:** **Plasmids used in this study**.

**Name**	**Relevant description**	**References**
pPl2	base for the construction of reporter strains	Lauer et al., 2002
pKSV-7-P*lmo2230*::*eGFP*	source of the SigB reporter construct	Utratna et al., 2012
pCG1	pPL2-P*lmo2230::eGFP*	this study
pCG8	pPL2-*Phly::eGFP*	this study
pAD-cGFP	used to make constitutively eGFP-expressing strain	Balestrino et al., 2010

Reporter fusions were constructed in pPL2, a site specific integration vector for *L. monocytogenes*. Specifically, an eGFP coding sequence codon-optimized for *L. monocytogenes* (Utratna et al., 2012) was used to construct two reporter fusions, including (i) a reporter fusion with a σ^B^-dependent promoter (P*lmo2230*) to assess σ^B^ activity and (ii) a reporter fusion with a PrfA-dependent promoter (P*hly*) to assess PrfA activity. The promoters used for the reporter constructs were selected to represent genes regulated as exclusively as possible by the respective transcription factors. *lmo2230*, which encodes a putative arsenate reductase, is σ^B^-regulated (Kazmierczak et al., 2003; Hain et al., 2008; Raengpradub et al., 2008; Oliver et al., 2009) and has been used previously in reporter systems for σ^B^ activity (Ondrusch and Kreft, 2011; Utratna et al., 2012). Although an early study suggested possible PrfA contributions to *lmo2230* regulation (Milohanic et al., 2003), subsequent studies (e.g., Ollinger et al., 2009) determined *lmo2230* to be solely σ^B^-regulated. Specifically, *lmo2230* is exclusively σ^B^-regulated under NaCl stress (Utratna et al., 2011). The virulence gene *hly* encodes the PrfA-regulated hemolysin listeriolysin O (LLO) (Mengaud et al., 1991; Milohanic et al., 2003; Ollinger et al., 2009).

The P*hly*::eGFP construct was created by amplifying the *hly* promoter (the first 204 bp upstream of the start codon, including a 123 bp 5′ untranslated region) from *L. monocytogenes* 10403S and the eGFP coding sequence from pKSV7-P*lmo2230*::*eGFP*, followed by a splicing by overlap-extension (SOE) PCR to join the two PCR products. The resulting PCR product was cloned into pPL2 creating pCG8 (Table 2). The P*lmo2230*::*eGFP* reporter was constructed using *E. coli* COB631 (obtained from Conor O'Bryne), which contains the plasmid pKSV7 with the *lmo2230* promoter (the first 443 bp upstream of the start codon, including a 110 bp 5′ untranslated region) fused to a codon-optimized version of eGFP (pKSV7-P*lmo2230*::*eGFP*) (Utratna et al., 2012); this reporter construct was subcloned into pPL2, creating pCG1 (Table 2). The inserts in the resulting plasmids were verified by sequencing (Sanger Sequencing Core facilities, Cornell University). Plasmid construction was performed in *E. coli* DH5α and final plasmid constructs were introduced into *E. coli* SM10 as a source strain for conjugation into *L. monocytogenes* 10403S. Presence of the construct in *L. monocytogenes* 10403S was confirmed by PCR with primers CG17 and CG18 (Supplementary Table 1) and by verifying fluorescence using a Zeiss (Jena, Germany) LSM 510 confocal microscope. As a negative control, the reporter constructs were cloned into the appropriate null mutant backgrounds, creating strains 10403S Δ*prfA*, P*hly*::eGFP and 10403S Δ*sigB*, P*lmo2230*::*eGFP* (Table 1). As *prfA* also has a σ^B^-dependent promoter (Freitag et al., 1993; Nadon et al., 2002; Ollinger et al., 2009), we cloned the PrfA reporter construct into a *sigB* null mutant background, creating strain 10403S Δ*sigB*, P*hly*::*eGFP* (Table 1), to investigate the influence of σ^B^ on PrfA activity.

To create constitutively GFP-expressing *L. monocytogenes*, pAD-cGFP was obtained from Balestrino et al. (2010), introduced into *E. coli* SM10 and conjugated into *L. monocytogenes* 10403S.

### Salt and heat stress experiments

Stress exposure experiments were performed with bacteria grown, as described above, to either log or stationary phase. The σ^B^ and PrfA reporter strains reached the desired OD_600_ at the same rate as the parent and the constitutively GFP expressing control strains during growth of log and stationary phase cultures, indicating no growth defect associated with the reporter construct or the constitutive expression of GFP during growth in BHI at 37°C (Supplementary Figure 1). To compensate for potential differences between the strains during stress exposure, all analyses were performed based on the proportion of cells that were GFP positive within one strain.

All stress exposure experiments were performed either in phosphate buffered saline (PBS) to simulate nutrient-depleted conditions or in a nutrient-rich medium (BHI). *L. monocytogenes* survives well in PBS, showing a limited increase in bacterial numbers over 200 min (<1 log). All stress exposure experiments were conducted in 4 ml volumes using 16 by 125 mm disposable borosilicate glass tubes incubated with aeration (shaking at 200 rpm). Log or stationary phase cultures were used directly for the heat stress experiments in BHI. For the heat stress experiments in PBS, 200 μl of log or stationary phase culture was added to 4 ml PBS, which had been pre-warmed to 37°C. For heat stress experiments, cultures were incubated at 45°C while controls were incubated at 37°C. For the salt stress experiments in BHI, 3 ml log or stationary phase culture was added to 1 ml BHI with 17.6% w/v NaCl for a final concentration of 4.4% w/v, pre-warmed to 37°C; for the negative control, 3 ml culture was added to 1 ml BHI without added salt, pre-warmed to 37°C. For the salt stress experiments in PBS, 200 μl log or stationary phase culture was added to 4 ml pre-warmed (37°C) PBS with 4.6% w/v NaCl (final concentration 4.4% w/v); the negative control was 200 μl of culture added to 4 ml PBS without the added salt. Standard PBS contains 0.8% NaCl, while standard BHI contains 0.5% added NaCl. While we recognize that additional NaCl may originate from the tissue components inherently present in BHI, we opted to add the same final exogenous concentration of NaCl to both PBS and BHI.

### Flow cytometry analysis

Flow cytometry (FCM) analysis of the reporter fusion strains exposed to different stress conditions was performed on cells collected right before (*t* = 0) or at 10, 20, 30, 60, 100, 150, and 200 min after initiation of salt or heat stress exposure. Cells collected at these time points were fixed in 1% paraformaldehyde and counter-stained with wheat-germ agglutinin conjugated to Alexa Fluor 633 (Invitrogen). For each experimental run, a sample of each culture was microscopically confirmed to be in single cell suspension; samples that did not meet this criterion were vortexed vigorously and reassessed. A confirmed sample was then diluted 1:400 in PBS and 50,000 individual cells were analyzed on a BD (San Jose, CA) FACS Aria Fusion flow cytometer to determine their eGFP status (positive/negative). Bacteria were gated on Alexa Fluor 633 so that no more than 0.1% of negative control bacteria (*L. monocytogenes* 10403S parent strain) and none of the PBS background registered as positive. Finally, the level of green spectrum autofluorescence in *L. monocytogenes* was determined by running an eGFP-negative parent strain control sample. The cutoff for eGFP was set so that no more than 1.5% of this negative control registered as eGFP positive. A strain constitutively expressing GFP was used as a positive control. All experiments were performed in at least three independent biological replicates (i.e., bacterial cells were grown and collected on different days).

### Confocal microscopy

Confocal microscopy was performed to visualize the eGFP fluorescence of individual cells of the PrfA and σ^B^ reporter strains. Bacteria were collected after 200 min of heat stress in PBS and fixed in 1% PFA as described above. A 5 μl aliquot of the fixed cell suspension was streaked on a glass slide and a coverslip was mounted with glycergel (Invitrogen). Images were taken on a Zeiss LSM510 confocal microscope with a 63X objective.

### RT-qPCRs

Reverse transcriptase (RT)-qPCR was used to: (i) confirm results from the reporter fusion experiments at the RNA level; and to (ii) assess the stability of the P*hly::eGFP* and P*lmo2230::eGFP* reporter mRNAs at 37 and 45°C. For all RT-qPCR experiments, cDNA was obtained from bacterial cells using a protocol previously detailed (Tang et al., 2015) with minor modifications. Briefly, 100 μl of a 1:10 mixture of acid-phenol chloroform in 100% EtOH was added to the bacterial cultures for each ml of culture. The cells were pelleted and lysed in lysozyme (20 mg/ml) and proteinase K (4 mg/ml) for 30 min, followed by bead-beating in Tri-Reagent (Life Technologies, Carlsbad CA), phase-separation with 1-bromo-3-chloropropane, and precipitation in isopropanol overnight at −80°C. The RNA pellet was then washed in 75% EtOH followed by treatment with Ambion Turbo DNAse and a second phenol-chloroform extraction, followed by overnight precipitation in 100% EtOH. The RNA pellet was washed in 70% EtOH and 500 ng of RNA was transcribed to cDNA using TaqMan® Reverse Transcription Reagents (Invitrogen Catalog Number N8080234) as per the manufacturer's manual. qPCRs were performed with SybrGreen (Invitrogen), with two technical replicates per sample, in optical 96-well plates on an ABI Prism 7,300 system (Applied Biosystems, Foster City, CA) using: (i) one cycle at 95.0°C for 10 min; (ii) 40 cycles at 95.0°C for 15 s and 55.0°C (except for *eGFP* PCRs, which used 60°C) for 1 min; and (iii) a dissociation curve. Genomic DNA isolated from *L. monocytogenes* 10403S was used to generate standard curves to determine optimal annealing temperatures of 55° or 60°C for *eGFP* and the efficiencies of the primer pairs used (i.e., primers targeting *eGFP, prfA, sigB, hly, lmo2230*, and *rpoB*). Serial dilutions were made to yield 10, 10^5^ and 10^7^ template copy numbers per reaction. The slope of the standard curve was determined and primer efficiency e was calculated as *e* = 2^−^^1/slope^ (Supplementary Table 1).

RT-qPCR was used to confirm the results from the reporter fusion experiments performed on log phase cells exposed to heat stress in PBS. For these experiments, 3 ml of log phase culture was added to 7 ml PBS pre-warmed to 45°C (heat exposed) or 37°C (control), followed by incubation for 5, 20, or 40 min at either 45°C or 37°C. cDNA was isolated from cells collected at these time points and RT-qPCR was performed (as described above) to determine the transcript levels for *prfA, sigB, hly* and *lmo2230*. The fold change (fc) in transcript levels between heat exposed and control cells was calculated using the following formula where *e* is the efficiency of the respective target primers (see Supplementary Table 1): *FC* = $\frac{e{\left(target\right)}^{ct\left(target,\;heat\;treated\right)\;-\;ct\left(target,\;control\right)}}{e{\left(rpoB\right)}^{ct\left(rpoB,\;heat\;treated\right)\;-\;ct\left(rpoB,\;control\right)}}$. The reported values represent the means of three independent biological replicates.

To assess the stabilities of the P*hly::eGFP* and P*lmo2230::eGFP* reporter mRNAs at 37 and 45°C, 5 ml of log phase cells in BHI were incubated at either: (i) 45°C for 30 min to ensure strong induction of the *eGFP* mRNA; or (ii) 37°C for 30 min (as control). At this time, an initial sample of cells was collected (*t* = 0) and transcription was stopped by adding rifampicin (Invitrogen) to a final concentration of 50 μg/ml. Additional samples were collected at *t* = 5 and *t* = 20 min after the addition of rifampicin. mRNA isolation, cDNA preparation, and qPCR were performed as detailed above. The fraction of the mRNA remaining (f) at each time point was calculated using *f* = 2^(*ct eGFP*(*t* = 0) − *ct eGFP*(*t* = *x*))^, where ct eGFP(*t* = 0) is the *ct*-value at *t* = 0 and ct eGFP(*t* = x) the *ct*-value at *t* = 5 or 20 (Archambault et al., 2013). As our analyses were performed to define the decay rate, the *ct*-values for eGFP were not normalized to *rpoB*. The reported values represent the means of two independent biological replicates, each performed at 37° and 45°C, resulting in four data points per reporter.

### Data analysis

All statistical analyses were performed in R version 3.2.1 (R Core Team, 2015), using the packages lme4 version 1.1-12 (Bates et al., 2014), lmerTest version 2.0-32 (Kuznetsova et al., 2016), ggplot2 version 2.1.0 (Wickham, 2009), and dplyr version 0.5.0 (Wickham, 2011). For FCM-based data, analyses were performed using the proportion of 50,000 cells per sample that were eGFP positive as the dependent variable. The data were logit transformed (Warton and Hui, 2011) and separate linear models were fitted, using lme4, for each heat and salt stress data immediately before exposure to heat or salt stress (*t* = 0) and after 10, 20, 30, 60, 100, 150, and 200 min of stress exposure. Model selection was by AIC-based backwards stepwise removal process, beginning with a full model up to three-way interactions. Comparisons between (i) stress exposed cells and their controls and (ii) the wildtype strain and its isogenic Δ*sigB* mutant were made at *t* = 0, 60, 100, 150, 200 using lsmeans. Holm-Bonferroni-corrected *p*-values and an α = 0.05 were used as cutoff for statistical significance. The modeled logit predictions along with their standard errors and confidence intervals were back-transformed into probability values for plotting and discussion.

For the mRNA stability data, analyses were performed on the absolute *ct*-values. With exponential decay, these vary linearly over time. A linear model was fitted to the slopes of the mRNA decay using the lm function in R (R Core Team, 2015). The slopes were compared using lsmeans to obtain a Tukey-corrected *p*-value (averaged over temperature) with a cutoff of α = 0.05 for significance.

For mRNA induction data, a mixed effect linear model was fitted on the fold change, with a full three-way interaction between reporter, strain, and time effects. Fold change was log-transformed. To determine whether individual mRNA levels were increased (indicated by a fold change significantly larger than 1) after exposure to 45°C compared to 37°C at each time point, lsmeans was used to perform a one-sided effect test, with Holm-Bonferroni-corrected *p*-values and an α = 0.05 as cutoff for significance. To determine whether σ^B^ influenced *prfA* and *hly* transcript levels, comparisons between fold changes in transcript levels in the wt and Δ*sigB* mutant strain background at individual time-points (*t* = 5, 20, 40 min) were performed using lsmeans with Holm-Bonferroni-corrected *p*-values and α = 0.05 as cutoff for significance.

## Results

### Sigma B and PrfA activity are induced stochastically at the single cell level

To visualize the activities of σ^B^ and PrfA in individual *L. monocytogenes* cells, we constructed reporter fusions between promoters that are activated by either σ^B^ or PrfA and a gene encoding eGFP that had been codon-optimized for *L. monocytogenes*. Initial evaluation of the reporter fusion constructs by confocal microcopy showed that (i) the σ^B^ reporter strain (10403S::P*lmo2230*-eGFP) emitted fluorescence under conditions known to induce σ^B^ activity (i.e., stationary phase) and (ii) the PrfA reporter strain (10403S P*hly::eGFP*) emitted strong fluorescence in the presence of active PrfA (e.g., after 45°C heat exposure). Negative controls verified that fluorescence was induced exclusively by σ^B^ or PrfA, respectively, for *lmo2230* and *hly*; the Δ*sigB* and Δ*prfA* strains containing reporter fusions (10403S Δ*prfA*, P*hly::eGFP* and 10403S Δ*sigB*, P*lmo2230::eGFP*) did not show any fluorescence. For the PrfA eGFP reporter, a positive control was possible; transformation of the P*hly::eGFP* construct into a strain carrying a mutation that constitutively activates PrfA (PrfA^*^ G155S) (Shetron-Rama et al., 2003; Ollinger et al., 2009) resulted in eGFP fluorescence levels comparable to those of a control strain constitutively expressing GFP (Supplementary Figure 2). Creation of a positive control for the σ^B^ reporter strain is not possible, as strains that constitutively express σ^B^ grow poorly or die (Conor O'Byrne, personal communication).

**Figure 1:**
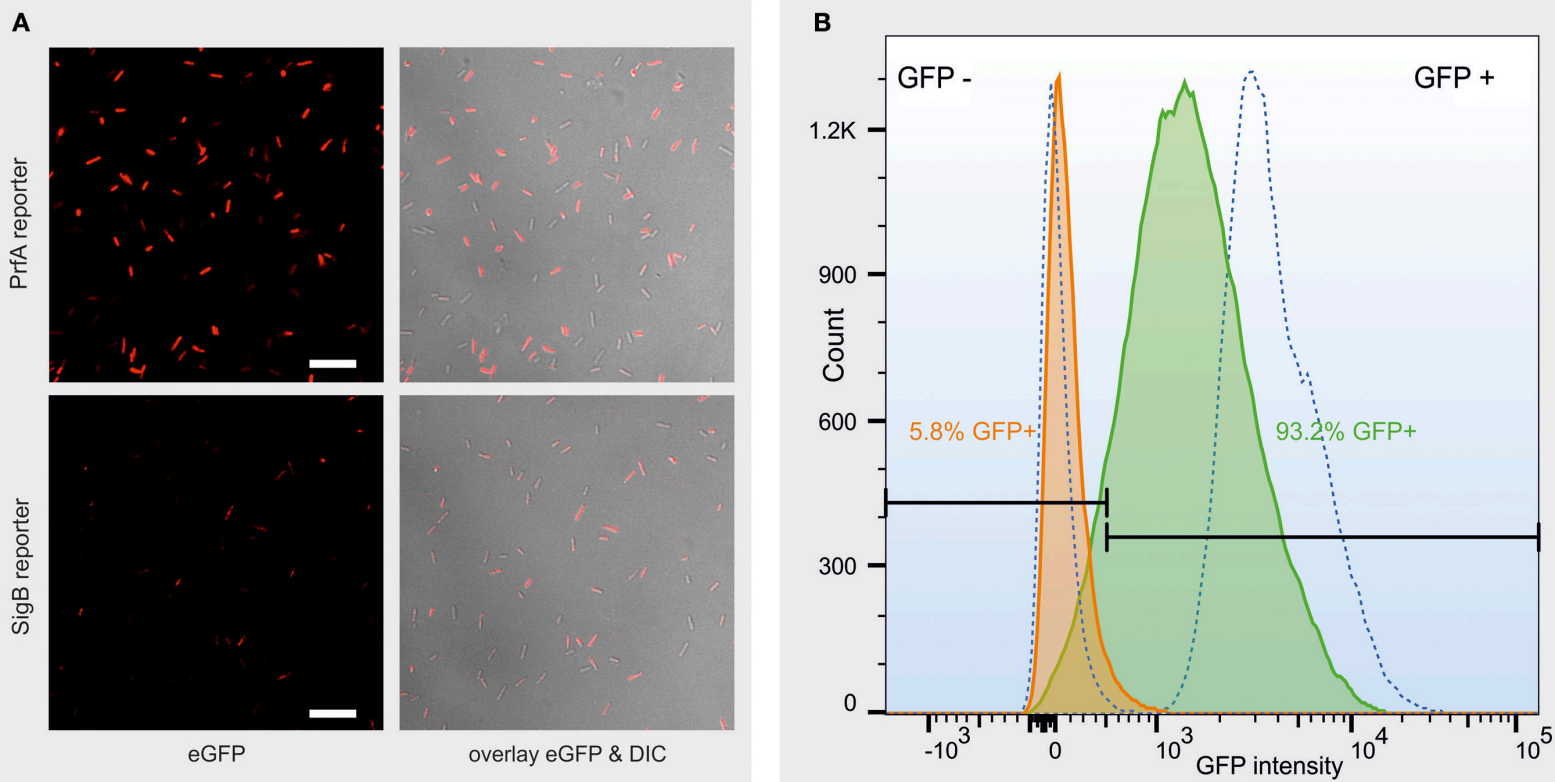
**(A)** Representative confocal images of a log phase PrfA reporter strain culture (top row) and a log phase σ^B^ reporter strain culture (bottom row) after 200 min exposure to heat stress in PBS. Left column: eGFP fluorescence, false-colored red for better contrast. Right column: overlay of the eGFP fluorescence with the differential interference contrast (DIC) image. Scale bar = 10 μm. **(B)** Representative fluorescence histogram from FCM data measuring the eGFP status of individual cells of a log phase PrfA reporter strain *L. monocytogenes* 10403S culture, after 100 min incubation in PBS at either 37°C (orange curve) or 45°C (green curve). 10403S wt (left dotted curve) and constitutive GFP (right dotted curve) are plotted as non-fluorescent and strongly fluorescent controls, respectively. GFP ± gates (black, horizontal bars) were set so that no more than 1.5% of the non-fluorescent controls is included in the GFP+ gate. The proportion of GFP positive cells is presented in the color that matches the curve that the data represent. The *x*-axis represents GFP intensity in arbitrary units, measured as GFP-area, in log scale.

Confocal microscopy on *L. monocytogenes* log phase cells exposed to heat stress in PBS showed that both cells containing the σ^B^ or the PrfA reporter construct were heterogeneous with regard to eGFP expression, with some cells fluorescing strongly while others do not fluoresce at all (Figure 1A). Analysis of the reporter fusion strains by FCM showed a relatively broad peak, indicating various levels of eGFP fluorescence that fluctuated between “many cells off” to “many cells on” in response to heat and salt stress for both σ^B^ and PrfA, never fully reaching either 0 or 100% cells in either state. Representative FCM data are shown in Figure 1B, illustrating that log phase PrfA reporter cells were predominantly eGFP negative (5.8% eGFP positive) when incubated at 37°C in PBS for 100 min, while a majority of cells was eGFP positive after incubation at 45°C in PBS for 100 min (93.2% eGFP positive). Hence, both confocal microscopy and flow cytometry show that even under a single condition, *L. monocytogenes* cells are heterogeneous with regard to PrfA or σ^B^ activity, illustrating an inherent level of noise in the activities of PrfA and σ^*B*^ resulting from stochastic responses to changes in the environment. Interestingly, across all tested conditions, a significantly larger proportion of cells showed active PrfA than they did active σ^B^. For example, when averaging across both growth phases (log and stationary phase) and media (BHI and PBS), after 200 min of heat stress, 55% of PrfA reporter cells, but only 5.5% of σ^B^ reporter cells, were eGFP positive (*p* < 0.0001).

### The proportion of cells with active PrfA increased in response to heat stress in a growth-phase dependent manner

**Figure 2:**
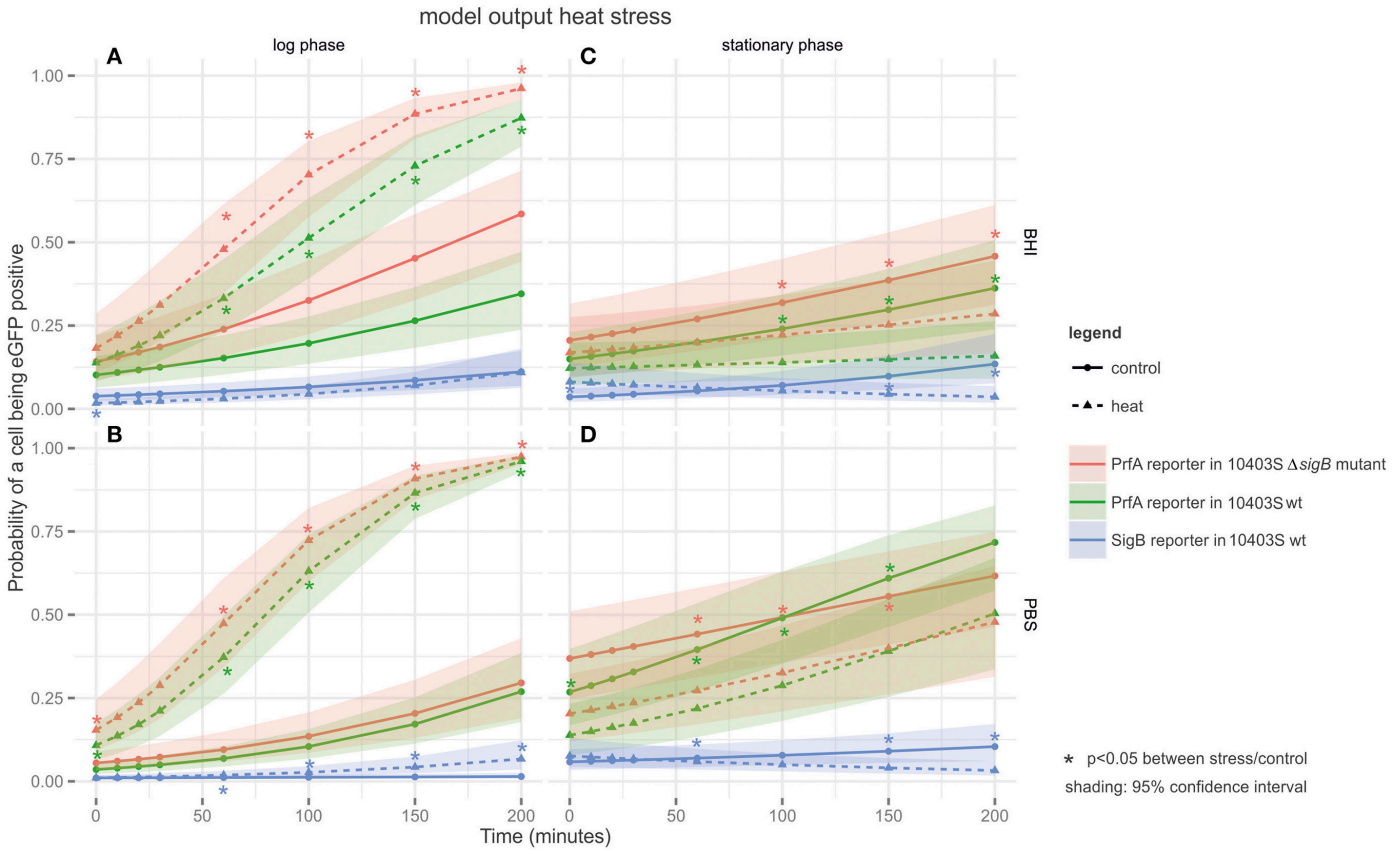
**Output of the linear model fitted to the heat FCM data**. The panels are tiled to represent cultures grown to different growth phases in either BHI or PBS: **(A)** log phase cultures in BHI; **(B)** log phase cultures in PBS; **(C)** stationary phase cultures in BHI; and **(D)** stationary phase cultures in PBS. The y-axis represents the probability of a cell being eGFP-positive (as a result of active PrfA or σ^B^), while the x-axis represents time in min. Three different strains are plotted on the graphs: the PrfA-reporter construct in a *L. monocytogenes* 10403S background, the PrfA-reporter construct in a *L. monocytogenes* 10403S Δ*sigB* mutant background, and the σ^B^-reporter construct in a *L. monocytogenes* 10403S background. Dotted lines represent data collected under the stress condition (45°C heat), the solid line represents the control condition (37°C), the shading around each line represents the 95% confidence interval. ^*^Are color coded to match the strain they pertain to and indicate a significant difference in the probability that a cell is eGFP positive in the stress condition vs. the control condition within one strain at one time point.

PrfA activity in response to heat stress was monitored at the single cell level under four different conditions: log phase cells in a rich medium (BHI) (Figure 2A) or in PBS (Figure 2B), and stationary phase cells in a rich medium (BHI) (Figure 2C) or in PBS (Figure 2D). A cell was defined as having active PrfA if it expressed eGFP fluorescence above the autofluorescence of the parent *L. monocytogenes* strain that does not contain a reporter fusion construct. Log phase cells exposed to heat in BHI showed significantly higher proportions of cells with active PrfA (at *t* = 60, 100, 150, and 200; Figure 2A) compared to the controls incubated at 37°C for both the PrfA reporter strain and its isogenic Δ*sigB* mutant. The same pattern of heat induction was observed for log-phase cells in PBS (Figure 2B). For example, PrfA activity was observed in 95.4% of log phase cells exposed to heat (at *t* = 200 min in PBS) compared to 24.7% in the respective control (*t* = 200, PBS, 37°C), *p* < 0.001.

For stationary phase cells in BHI, no significant activation of PrfA by heat was observed. Stationary phase cells exposed to 45°C showed significantly lower proportions of cells with active PrfA at *t* = 100, 150, and 200 (Figure 2C) compared to the 37°C control condition for both the PrfA reporter and its isogenic Δ*sigB* mutant. For example, PrfA activity was observed in 15.6% of heat exposed stationary phase cells (at *t* = 200 in BHI) compared to 37.9% in the respective control (*t* = 200 in BHI at 37°C), *p* < 0.001. For stationary phase cells in PBS, the same pattern was observed (Figure 2D).

Across all heat stress conditions (Figure 2), there was no significant difference in the proportion of cells with active PrfA when comparing the 10403S PrfA reporter strain and its isogenic Δ*sigB* mutant, suggesting no significant additional contributions of σ^B^ to PrfA activity under the conditions studied.

### The proportion of cells with active PrfA increased in log phase cells exposed to salt stress under nutrient limiting conditions

**Figure 3:**
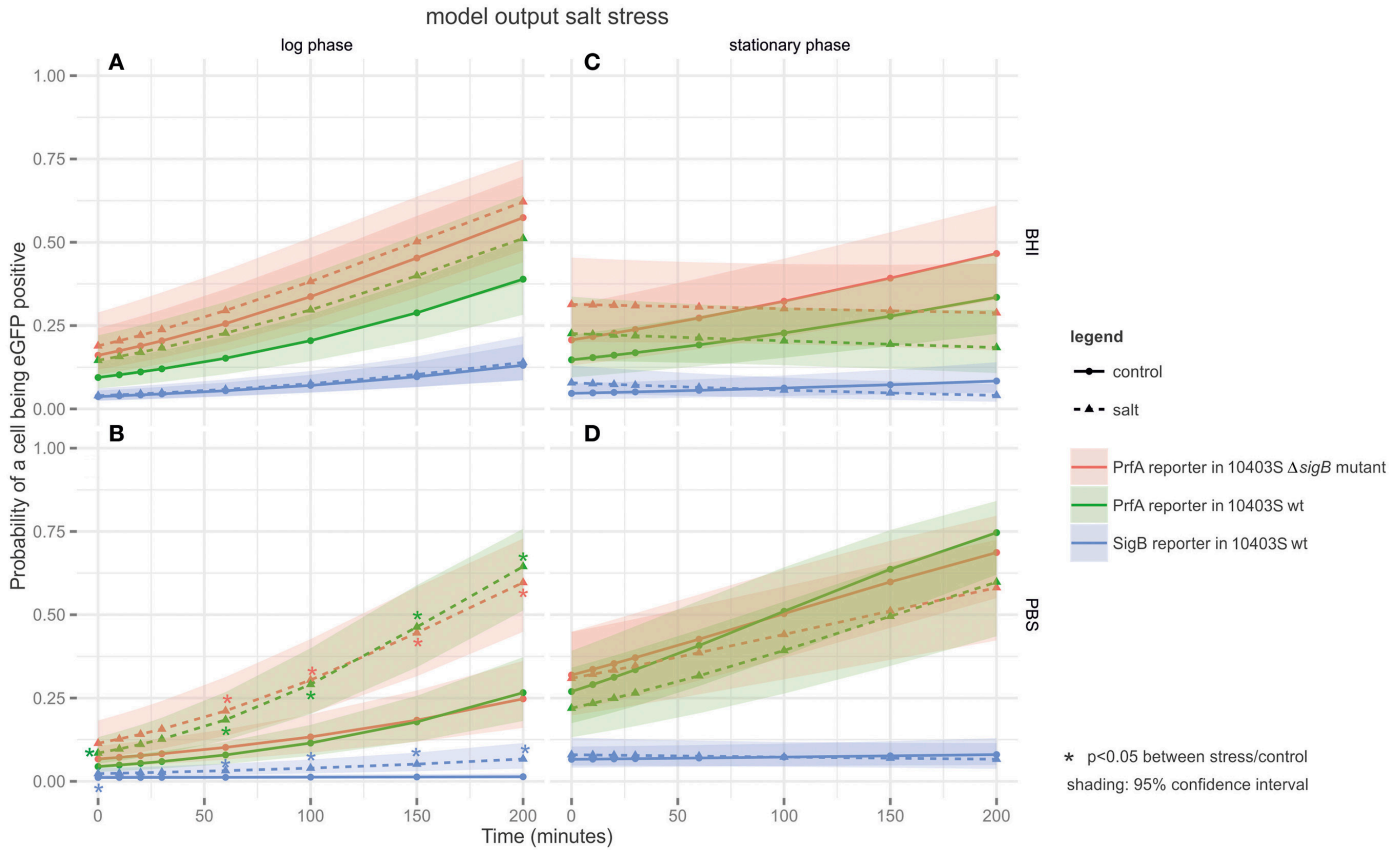
**Output of the linear model fitted to the salt stress FCM data**. The panels are tiled to represent cultures grown to different growth phases in either BHI or PBS: **(A)** log phase cultures in BHI; **(B)** log phase cultures in PBS; **(C)** stationary phase cultures in BHI; and **(D)** stationary phase cultures in PBS. The y-axis represents the probability of a cell being eGFP-positive (as a result of active PrfA or σ^B^), while the x-axis represents time in min. Three different strains are plotted on the graphs: the PrfA-reporter construct in a *L. monocytogenes* 10403S background, the PrfA-reporter construct in a *L. monocytogenes* 10403S Δ*sigB* mutant background, and the σ^B^-reporter construct in a *L. monocytogenes* 10403S background. Dotted lines represent data collected under the stress condition (4.4% salt stress), the solid line represents the control condition (no salt), the shading around each line represents the 95% confidence interval. ^*^Are color coded to match the strain they pertain to and indicate a significant difference in the probability that a cell is eGFP positive in the stress condition vs. the control condition within one strain at one time point.

For log phase cells grown in BHI, exposure to salt (4% NaCl) only had a mild effect on PrfA activity; log phase cells exposed to salt in BHI (Figure 3A) showed no significant difference in the proportions of cells with active PrfA compared to the controls without the added salt. In contrast, log phase cells exposed to salt in PBS (Figure 3B), showed significantly higher proportions of cells with active PrfA at all time points in both the PrfA reporter and its isogenic Δ*sigB* mutant. For example, PrfA activity was observed in 69.5% of salt exposed log phase cells (at *t* = 200 in PBS) compared to 30.8% in the respective control conditions (*t* = 200, PBS, no salt, *p* < 0.001).

Stationary phase cells exposed to salt stress in BHI (Figure 3C) showed no significant difference in the proportions of cells with active PrfA compared to the controls without the added salt for both the PrfA reporter and its isogenic Δ*sigB* mutant. Therefore, exposure to salt stress did not induce PrfA activity in this condition. The same pattern was observed in stationary phase cells exposed to salt in PBS (Figure 3D). For example, PrfA activity was observed in 54.2% of salt exposed stationary phase cells (at *t* = 200 in PBS) compared to 54.5% in the respective control (*t* = 200, PBS, no salt, *p* = 0.71).

Across all salt stress conditions (Figure 3), there was no significant difference in the proportion of cells with active PrfA when comparing the PrfA reporter to its isogenic Δ*sigB* mutant.

### Heat stress lead to a significant increase in the proportion of cells with active σ^B^ in log phase cells in PBS, but not in log phase cells grown in BHI or in stationary phase cells

σ^B^ activity after heat stress was measured under the same conditions used to assess PrfA activity: log phase cells in a rich medium (BHI) (Figure 2A) or in PBS (Figure 2B), and stationary phase cells in a rich medium (BHI) (Figure 2C) or in PBS (Figure 2D). A cell was defined as having active σ^B^ if it expressed eGFP fluorescence above the autofluorescence of the parent *L. monocytogenes* strain. Across conditions, the proportion of cells with active σ^B^ was markedly lower than those with active PrfA (Figure 2).

Log phase cells exposed to 45°C in BHI (Figure 2A) showed no significant difference in the proportion of cells with active σ^B^ compared to the controls incubated at 37°C. However, log phase cells exposed to 45°C heat stress in PBS (Figure 2B) showed significantly higher proportions of cells with active σ^B^ at all time-points compared to the 37°C controls. For example, σ^B^ activity was observed in 3.7% of heat exposed log phase cells (at *t* = 200 in PBS) compared to 0.7% in the respective control (*t* = 200, PBS, 37°C, *p* < 0.001).

In stationary phase cells, heat stress did not induce σ^B^ activity in BHI (Figure 2C) or in PBS (Figure 2D). In fact, stationary phase cells exposed to 45°C heat in both media (Figures 2C,D) showed significantly lower proportions of cells with active σ^B^ (at *t* = 60, 150, and 200) compared to the 37°C controls. For example, σ^B^ activity was observed in 2.7% of heat exposed stationary phase cells (at *t* = 200 in BHI) compared to 13.5% in the respective control (*t* = 200, BHI, 37°C, *p* < 0.001).

### Salt stress increased the proportion of cells with active σ^B^ in log phase cells in PBS, but not under any of the other tested conditions

σ^B^ activity in response to salt stress was monitored under the same conditions as for heat stress (Figure 3). In log phase cells exposed to salt (4% NaCl) in BHI, the salt exposed cells did not show significantly higher proportions of cells with active σ^B^ compared to the controls without the added salt (Figure 3A). However, log phase cells exposed to salt in PBS (Figure 3B) showed significantly higher proportions of cells with active σ^B^ at all-time points (*t* = 60, 100, 150, 200) compared to the controls without the added salt. For example, σ^B^ activity was observed in 8.4% of salt exposed log phase cells (at *t* = 200 in PBS) compared to 2.6% in the respective control (*t* = 200, PBS, no salt, *p* < 0.001).

In stationary phase cells exposed to salt stress in BHI (Figure 3C), the proportion of cells with active σ^B^ did not increase compared to the controls without the added salt. For example, σ^B^ activity was observed in 7.5% of salt exposed stationary phase cells (at *t* = 200 in BHI) compared to 14.4% in the respective control (*t* = 200, BHI, no salt, *p* = 0.091). Stationary phase cells exposed to salt in PBS (Figure 3D) showed no significant changes in the proportions of cells with active σ^B^.

### RT-qPCR shows no difference in the stability of PrfA reporter fusion mRNA compared to σ^B^ reporter fusion mRNA

As the FCM data showed that generally a much larger proportion of cells had active PrfA compared to the proportion of cells that had active σ^B^ (e.g., in log phase cells exposed to 45°C heat for 200 min in PBS, on average, 95.5% showed active PrfA while 3.7% of cells showed active σ^B^), we performed RT-qPCR experiments to assess the relative mRNA stability of the PrfA and σ^B^ reporter fusions, using qPCR primers targeting eGFP. Specifically, we hypothesized that the PrfA reporter construct (P*hly*::*eGFP*) may be more stable than the σ^B^ reporter construct due to a longer native 5′ untranslated region (UTR) (123 nt) in this construct as compared to a shorter (51 nt) 5′ UTR in the σ^B^ reporter construct (P*lmo2230::eGFP*). qPCR data showed a clear reduction of mRNA levels for both reporter constructs after rifampicin treatment to arrest transcription. The data fit a general linear model that allowed us to test whether (i) temperature and (ii) the choice of reporter promoter including its 5′UTR (i.e., P*lmo2230::eGFP* as reporter for σ^B^ activity and P*hly::eGFP* as reporter for PrfA activity) had a significant effect on mRNA decay (which measures RNA stability); a *p*-value of 0.25 for the effect of reporter gene on RNA decay showed that there was no significant difference in mRNA stability between the two reporter genes (Supplementary Figure 3).

### RT-qPCR supports differences in PrfA and σ^B^ activation by heat

**Figure 4:**
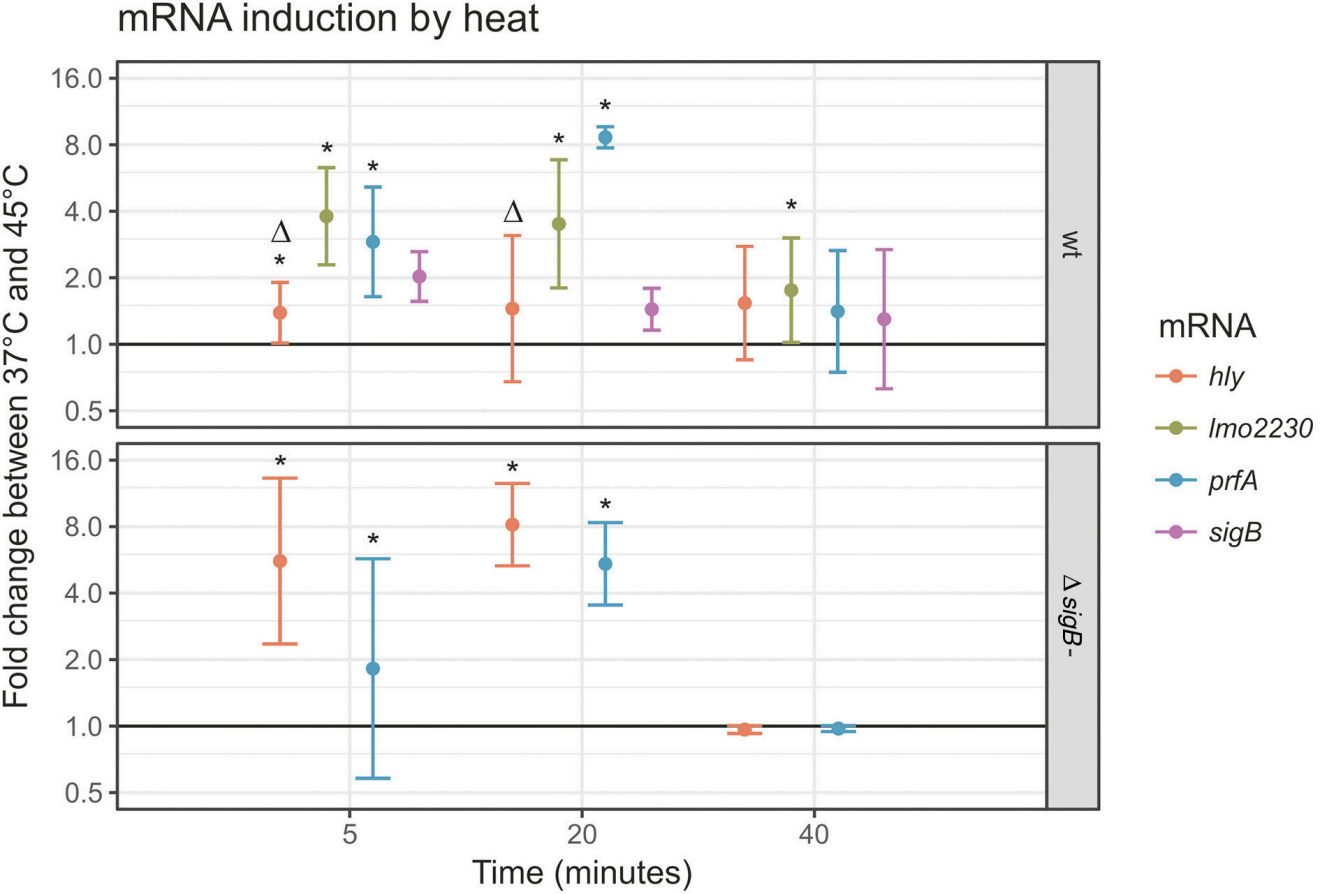
*****prfA, hly, sigB*** and ***lmo2230*** transcript levels in response to 45°C heat stress in log phase cells**. RT-qPCR was performed with primers for *prfA, hly, sigB* and *lmo2230* on log phase *L. monocytogenes* 10403S parent strain cultures (top panel) or its otherwise isogenic Δ*sigB* strain (bottom panel). The y-axis represents the log fold change between cells incubated in PBS at 37° or 45°C, determined by the delta-delta ct method. A value of 1 represents no change (horizontal line). For each time point, the average ± SD were plotted. ^*^Indicates a statistically significant fold change between 37° and 45°C for this mRNA at this time point (*p* < 0.05), Δ indicates a significant difference between the wt and the Δ*sigB* mutant strain at this time point (*p* < 0.05).

To confirm the results obtained with the reporter fusions, transcript levels for four genes (*prfA* and the PrfA-dependent *hly*; *sigB* and the σ^B^-dependent *lmo2230*) were determined by RT-qPCR in log phase cells exposed to heat stress in PBS (Figure 3). This condition was selected as our reporter fusion experiments indicated that it induced both PrfA and σ^B^ activity (Figure 2B). RT-qPCR data showed clear evidence for increased transcript levels for *prfA* after 45°C heat stress (2.9-fold increase at *t* = 5 and 8.5-fold increase at *t* = 20 compared to the 37°C controls, *p* < 0.001); Figure 4, top panel. By comparison, the PrfA-dependent *hly* showed only a mild increase in transcript levels after 45°C heat exposure (1.3-fold increase at *t* = 5 and 1.4-fold increase at *t* = 20 as compared to the 37°C control, *p* > 0.05). Overall, these data suggest that for the PrfA-mediated response to heat stress, an increase in *prfA* transcript levels is at least a part of the process. RT-qPCR data also showed evidence for mildly increased transcript levels for *sigB* after 45°C heat exposure (2.2 (*p* = 0.009) fold increase at *t* = 5 min, and 1.6 (*p* = 0.08) fold increase at *t* = 20 min compared to the 37°C controls). In contrast, the transcript levels for the σ^B^-dependent *lmo2230* increased in response to 45°C heat exposure (4.2-fold increase at *t* = 5 and 3.8-fold increase at *t* = 20 compared to the 37°C control, *p* < 0.001). Our finding that the fold change in transcript levels for the σ^B^-dependent *lmo2230* exceeds the fold change for *sigB* transcript levels is consistent with the fact that σ^B^ is post-translationally activated by a phosphorylation cascade involving anti-sigma factors (reviewed in O'Byrne and Karatzas, 2008). As a consequence, increased expression of the σ^B^ regulon does not necessarily require a parallel increase in *sigB* transcription levels.

To assess the effect of active σ^B^ on expression of the PrfA regulon, *prfA* and *hly* mRNA levels were also determined in the 10403S Δ*sigB* mutant strain (Figure 4, bottom panel). As expected, no *sigB* mRNA was detected in the 10403S Δ*sigB* mutant, and only traces of *lmo2230* RNA were detectable (absolute *ct* > 29 in all samples). In the parent strain, the fold change in *prfA* transcript levels in response to 45°C heat stress was numerically, but not statistically significantly higher than in the 10403S Δ*sigB* mutant strain, with the highest measurement at 8.5 vs. 5.2-fold change compared to the 37°C controls at *t* = 20 min; not significant at *p* = 0.34. In contrast, the *hly* transcript levels that depend on active PrfA showed a smaller increase in the parent strain after heat treatment than in the Δ*sigB* mutant strain (with the highest measurement at 1.4 vs. 8.2-fold change after 20 min, *p* < 0.001). These results reflect the known dual role for σ^B^ in PrfA regulation, including (i) positive regulation of *prfA* transcription (Nadon et al., 2002; Rauch et al., 2005; Schwab et al., 2005) and (ii) indirect negative regulation of PrfA activity (Ollinger et al., 2009).

## Discussion

Using reporter fusions that allow monitoring either PrfA or σ^B^ activity at the single cell level, we show the dynamic response of individual cells of *L. monocytogenes* to heat and salt stress. Our data provide a number of novel insights into the regulation of the activity of these two important transcriptional regulators, including evidence for their stochastic activation.

### PrfA and σ^B^ activity are induced stochastically

Differential and stochastic gene expression in clonal microbial populations allow for bet-hedging and labor-sharing within the population (Elowitz et al., 2002). Our data support that neither PrfA nor σ^B^ activity is induced by deterministic circuits that would turn these transcriptional regulators either “on” or “off” in all cells. Rather, confocal microscopy as well as FCM results presented here showed stochastic induction of PrfA and σ^B^ activity, which produced an inherent level of noise. As PrfA is essential for virulence and as σ^B^ contributes to resilience in *L. monocytogenes* (O'Byrne and Karatzas, 2008; De las Heras et al., 2011), it is conceivable that stochasticity in PrfA and σ^B^ activity is a mechanism of bet-hedging, creating sub-populations that are pre-adapted to enable survival under changing environmental conditions. While stochastic activation following salt stress has been demonstrated previously for σ^B^ (Utratna et al., 2012), our data provide novel insights into PrfA activation. Stochastic regulation of virulence gene expression has been demonstrated in other microbial species, including the *cap* genes in *S. aureus* (George et al., 2015), the type three secretion system 1 in *Salmonella* Typhi (Arnoldini et al., 2014), and flagella synthesis in *S. enterica* (Stewart and Cookson, 2014). The PrfA operon includes genes that encode secreted factors, e.g., the pore-forming toxin, LLO, which facilitates *L. monocytogenes* escape from the phagocytic vacuole in host cells, a mechanism that potentially lends itself to labor-sharing. However, the PrfA regulon also includes a number of genes that encode surface proteins (e.g., ActA, internalin A and B) for which advantages from labor-sharing are less obvious, as presumably each individual cell would need to express these proteins to successfully proliferate in the host (Gaillard et al., 1991). The necessity of PrfA-dependent surface proteins for successful host cell invasion thus may be a reason why a large proportion of individual *L. monocytogenes* cells were found to show active PrfA under some of the conditions in this study. A possible model is that stochastic expression of PrfA in a relatively small subpopulation of cells in a certain environment (e.g., outside a host or before initial invasion into host cells) provides for bet-hedging, while expression of PrfA in a majority of cells under other conditions (e.g., inside a host cell) reflects the importance of virulence gene expression during specific phases of infection. Future studies will be necessary to determine correlations among PrfA activity, σ^B^ activity and bacterial phenotype in terms of stress resistance, cell-cell interaction, growth, and host cell invasiveness.

### Heat and salt stress represent newly identified activator signals for PrfA

The reporter fusion data reported here show that the proportion of cells with active PrfA strongly increased after heat stress exposure of log-phase cells, but not in stationary phase cells. The same was true for the proportion of cells with active σ^B^, indicating the complexity of the underlying regulatory networks and the probable involvement of other, yet unknown regulatory factors that render the response to heat stress growth-phase dependent. We initially included a mild heat stress of 45°C to determine its effect on σ^B^ activity, since a Δ*sigB* mutant is less heat resistant than the wildtype and σ^B^ is involved in the heat shock response (Hu et al., 2007). Somewhat surprisingly, we found that in log phase cells after heat stress, the proportion of individual cells with active PrfA reached nearly 100%, which was higher than the proportion of cells with active σ^B^ under any conditions (the maximum observed was 14.8% of log phase cells with active σ^B^ after exposure to salt stress in BHI for 200 min). RT-qPCR confirmed heat induction of PrfA and showed an increase of *prfA* transcripts after 45°C heat exposure. While these data suggest that increased transcription of *prfA* may at least partially contribute to the increased levels of active PrfA observed after heat stress, the specific mechanism of 45°C heat stress induction of PrfA activity remains to be determined. PrfA activity is regulated at the transcriptional level through an autoregulatory feedback loop (Mengaud et al., 1991; Chakraborty et al., 1992) as well as at the post-transcriptional and post-translational levels, allowing for potential induction at various levels. A temperature-dependent post-transcriptional mechanism regulates *prfA* translation. The monocistronic *prfA* mRNA acts as a thermosensor by forming a temperature-dependent secondary structure in the 5′UTR that masks the ribosomal binding site at 30°C (Johansson et al., 2002), but not at 37°C, ensuring that PrfA-controlled virulence genes are expressed at typical mammalian host body temperatures. It is thus tempting to speculate that increases in temperatures above 37°C may further enhance *prfA* translation with subsequent enhanced *prfA* transcription through the auto-regulatory feedback loop. Alternatively, 45°C heat stress may also contribute to post-translational activation of PrfA. A previous study (Herbert and Foster, 2001) found no effect of a *prfA* null mutation on heat resistance. While 45°C may not be encountered frequently by *L. monocytogenes* during infection, heat stress, in general, could be encountered by *L*. *monocytogenes* during transmission, including in food processing-associated environments (e.g., hot water cleaning), foods (e.g., sublethal heat treatment), and, potentially, in certain hosts with higher body temperatures, e.g., chickens (Speer, 2006).

Alternatively, increased activity at 45°C may simply result from the complex regulation of PrfA activity, which is not fully understood, but appears to involve transcriptional upregulation mediated by the presence of branched chain amino acids (sensed via CodY; Lobel et al., 2015) and glucose-1-phosphate carbohydrate (via the mannose-type phosphotransferase systems; Ake et al., 2011). Furthermore, the presence of glutathione (produced by a *Listeria* glutathione synthase) also appears to activate PrfA (Reniere et al., 2015).

Our data also indicated that exposure of log phase cells to salt stress increased the proportion of cells with active PrfA, albeit this induction was considerably less pronounced than that observed in response to heat stress. While previous studies have shown that a *prfA* null deletion had no effect on *L. monocytogenes* survival of 11% salt stress (Ribeiro et al., 2014), an increase in osmolarity may serve as a signal for the imminent encounter of the pathogen with host cells in the duodenum, where the osmolarity is higher than in previously encountered gastrointestinal compartments due to the breakdown of gastric contents into smaller molecules. Therefore, increased osmolarity may represent an environmental signal that is used by *L. monocytogenes* to sense its presence in a host environment where induction of PrfA activity is needed to facilitate invasion of host cells. Whether results obtained in liquid cell culture media, such as in our experiments, translate to *L. monocytogenes* growing on food matrices, and particularly on solid ones, remain to be determined.

PrfA activity was not significantly altered in a Δ*sigB* mutant compared to the parent strain, although an intact σ^B^ contributed to higher *prfA* mRNA levels under heat stress. Thus, our reporter fusion data reported here further support the previously reported (Nadon et al., 2002; Ollinger et al., 2009) dual role of σ^B^ in regulating *prfA* transcription and expression of the PrfA regulon, including (i) σ^B^-dependent upregulation of *prfA* transcription through the prfAP1 promotor and (ii) σ^B^-dependent downregulation of PrfA-dependent genes (including *hly*) in the presence of active PrfA through a yet-to-be determined mechanism. The positive influence of σ^B^ on *prfA* transcription is supported by our RT-qPCR data showing higher *prfA* transcript levels in the parent strain compared to the Δ*sigB* mutant. The indirect negative influence of σ^B^ on PrfA activity is visible in the reporter fusion data that consistently showed a trend of higher PrfA activity in a *sigB* null background (Figure 2). This conclusion is further supported by RT-qPCR data, which showed lower transcript levels of the PrfA dependent *hly* in the parent strain compared to the Δ*sigB* mutant under heat stress (Figure 4). Future experiments with a double reporter strain for PrfA and σ^B^ activity may provide an opportunity to gain higher-resolution insights into the relation between PrfA and σ^B^ activity and to investigate the overlap between cells with high PrfA and σ^B^ activity.

### Heat and salt stress act as activators of σ^B^ under select conditions

Our data showed that heat stress exposure of log phase cells in PBS led to a significantly increased proportion of cells with active σ^B^; this effect was not seen for log phase cells grown in BHI or for stationary phase cells. Previous studies suggest that σ^B^ is involved in the heat stress response; σ^B^ has been shown to directly or indirectly regulate expression of several heat shock proteins, such as ClpP, and regulators of heat survival, including CtsR and HrcA (Hu et al., 2007). For example, an *hrcA* mutant was significantly less heat resistant than its parent strain and a σ^B^-dependent promoter was computationally identified upstream of *hrcA* (Hu et al., 2007). However, results from studies that investigated the impact of *sigB* null deletions on heat stress survival have varied from minor (Ferreira et al., 2001) to significant effects (Hu et al., 2007; Somolinos et al., 2010; Ait-Ouazzou et al., 2012). σ^B^ activity is governed by a complex regulatory network and is affected by multiple factors including nutrients and temperature (Guariglia-Oropeza et al., 2014), therefore different outcomes across studies likely reflect the different conditions, strains and media used. Overall, we provide further support that σ^B^ contributes to heat stress response in *L. monocytogenes*.

Our data showed an increased proportion of log phase cells with active σ^B^ in response to salt stress in PBS (but not in BHI), however, the proportion of cells with active σ^B^ did not exceed 15% in any of the tested conditions. By comparison, a study by Utratna et al. (2012) found about 42% of cells displaying σ^B^-dependent fluorescence after growing *L. monocytogenes* EGDe to log phase in 3.5% NaCl in BHI, compared to 13% with σ^B^-dependent fluorescence in our study. These differences could result from strain differences between 10403S and EGDe, but could also reflect different FCM instruments and protocols, or the different site in the genome where the reporter construct was integrated. Salt concentrations in the range of 0.5 M (2.9%) to 1.9 M (11%) have previously been shown to induce σ^B^ activity on a population level (Sue et al., 2003; Ringus et al., 2012) or to significantly inhibit growth of Δ*sigB* mutants (Abram et al., 2008; Ribeiro et al., 2014). Additionally, previous studies indicate that the type of osmotic stress used here strongly increases transcription of σ^B^-dependent genes (Sue et al., 2003, 2004). If σ^B^ activity is crucial for survival under salt stress, but a measurable increase in σ^B^ activity only occurs in a small proportion of cells, it is possible that the effect observed at the population-level results from increased σ^B^ activity in relatively few cells. This surprising finding could indicate either (i) a bet-hedging mechanism (as discussed in more detail below) or (ii) the existence of a mechanism for the whole population to profit from active σ^B^ in a proportion of cells in a labor-sharing fashion. Unlike extracytoplasmatic sigma factors such as σ^C^, σ^B^ is not secreted. However, one could speculate that a σ^B^-dependent, secreted factor may serve the whole population. While there are no known σ^B^-dependent proteins with an obvious potential for labor-sharing in *L. monocytogenes*, there are σ^B^-dependent factors produced by *L. monocytogenes* that do get secreted, including the products of *fri, sodA, lmo1068*, and *lmo0796* (Trost et al., 2005). Fri (*LMRG_0241*) is a homolog of the *E. coli dps* (DNA-binding protein from starving cells; Olsen et al., 2005) that prevents generation of hydroxyl radicals under anaerobic conditions by sequestering iron from Fenton reactions (Ilari et al., 2002). The σ^B^-dependent promoter upstream of *fri* (Polidoro et al., 2002) has been experimentally confirmed (Olsen et al., 2005). The superoxide dismutase encoded by *sodA* has a σ^B^-dependent promoter (Raengpradub et al., 2008) and is secreted by the auxiliary protein secretion system SecA (Archambaud et al., 2006). Much less is known about the functions of *lmo0796* (a *ycel*-family like protein) and the hypothetical protein *lmo1068*, both of which have been shown to be secreted (Trost et al., 2005) and to have σ^B^-dependent promoters (Hain et al., 2008; Oliver et al., 2010; Mujahid et al., 2013).

The observation that few individual cells induce σ^B^ activity to a detectable level, even in conditions known to strongly activate σ^B^, also has implications on the interpretation of transcriptional data generated at the population level (e.g., through microarrays). In any method that detects the cumulative signal from a population, it is possible that a relatively small number of cells with strong σ^B^ activity produce the signal while the remainder of cells stay “silent,” do not activate σ^B^ and hence do not induce transcription of the σ^B^ regulon. In summary, the following model could explain our observations for σ^B^ activity: generally, very few cells show high σ^B^ activity. Under conditions known to activate σ^B^, the number of cells with active σ^B^ increases, while a majority of cells still show undetectable levels of σ^B^ activity. It remains to be determined whether σ^B^ activity at levels below the detection limit of our assay can exert cellular functions, while keeping the “cost” (as discussed below) to the cell low.

### Induction of PrfA activity appears to be population-wide, while σ^B^ activity appears restricted to a minority subpopulation, indicating stronger bet-hedging, and/or labor sharing for σ^B^

Interestingly, we found that under all conditions evaluated, the proportion of individual cells that express detectable levels of active PrfA was significantly higher than for σ^B^. Other authors previously reported much higher levels of *prfA* mRNA compared to *sigB* mRNA in stationary phase cells (Olesen et al., 2010). The differences in proportion of cells with active PrfA and σ^B^ could indicate that it is advantageous to have a large number of cells with active PrfA under certain conditions, while it is advantageous for *L. monocytogenes* to activate σ^B^ in only a smaller subpopulation. Alternatively, our system may not be sensitive enough to pick up subtle changes in σ^B^ activity; if the effect of a transcription factor is amplified by a positive feedback loop, as with σ^B^, a relatively small number of active molecules can have a large effect. It is therefore possible that σ^B^ activity stays below the detection limit of our assay but still has an impact on the transcriptome. It is also possible that σ^B^ may be activated in large subpopulations, but under conditions other than those tested here.

The fact that the proportion of cells with active σ^B^ was found to be limited under stress conditions that have been shown previously to strongly induce σ^B^ activity is consistent with observations on the “fitness cost” of expressing active forms of σ^B^ and PrfA. Previous data clearly indicate that σ^B^ activity comes at a cost to the cell as supported by the fact that *L. monocytogenes* with either a *sigB* deletion or a deletion of regulators that activate σ^B^ show more rapid log phase growth as compared to wildtype strains (Chaturongakul and Boor, 2004). Also, attempts to create mutations with constitutively active σ^B^ have failed in the past or show very poor growth in *L. monocytogenes* (Conor O'Byrne, personal communication) as well as in *B. subtilis* (Redfield and Price, 1996). In contrast, constitutive activation of PrfA is possible and does not lead to growth deficiencies of comparable proportions. In fact, there are several known mutations that render PrfA constitutively active (PrfA^*^) (Ripio et al., 1997; Monk et al., 2008). Characterization of these mutants did not show any growth defects when grown in monoculture in rich media, however a fitness defect was detectable in competitive growth assays with the isogenic wildtype strains (Bruno and Freitag, 2010). This proposed model might help explain why σ^B^ activity is restricted to a relatively small number of individual cells compared to PrfA activity, resulting in stronger bet-hedging or labor-sharing in the case of σ^B^. We thus conclude that our data convincingly suggest that the dynamics and stochasticity of regulation of PrfA and σ^B^ activity differ at the single cell level.

## Author contributions

This study was conceived and designed by MW, KB, and CG. CG and SH conducted the labwork. Data analysis and interpretation was done by CG, DK, VG, MW. CG and MW wrote the manuscript with critical and crucial input by VG, KB, SH, and DK.

## Funding

This work was funded by a grant of the Swiss National Science Foundation to Claudia Guldimann (Grant No. P2BEP3_148881)

### Conflict of interest statement

The authors declare that the research was conducted in the absence of any commercial or financial relationships that could be construed as a potential conflict of interest.

## References

[B1] Abram F. Starr E. Karatzas K. A. Matlawska-Wasowska K. Boyd A. Wiedmann M.. (2008). Identification of components of the sigma B regulon in *Listeria monocytogenes* that contribute to acid and salt tolerance. Appl. Environ. Microbiol. 74, 6848–6858. 10.1128/AEM.00442-08 18806006 PMC2583506

[B2] Ait-Ouazzou A. Manas P. Condón S. Págan R. García-Gonzalo D. (2012). Role of general stress-response alternative sigma factors sigma(S) (RpoS) and sigma(B) (SigB) in bacterial heat resistance as a function of treatment medium pH. Int. J. Food. Microbiol. 153, 358–364. 10.1016/j.ijfoodmicro.2011.11.027 22177853

[B3] Ake F. M. Joyet P. Deutscher J. Milohanic E. (2011). Mutational analysis of glucose transport regulation and glucose-mediated virulence gene repression in *Listeria monocytogenes*. Mol. Microbiol. 81, 274–293. 10.1111/j.1365-2958.2011.07692.x 21564334

[B4] Allerberger F. Wagner M. (2010). Listeriosis: a resurgent foodborne infection. Clin. Microbiol Infect. 16, 16–23. 10.1111/j.1469-0691.2009.03109.x 20002687

[B5] Archambaud C. Nahori M. A. Pizarro-Cerda J. Cossart P. Dussurget O. (2006). Control of *Listeria* superoxide dismutase by phosphorylation. J. Biol. Chem. 281, 31812–31822. 10.1074/jbc.M606249200 16905535

[B6] Archambault L. Borchert J. S. Bergeron J. Snow S. Schlax P. J. (2013). Measurements of mRNA degradation in *Borrelia burgdorferi*. J. Bacteriol. 195, 4879–4887. 10.1128/JB.00659-13 23974029 PMC3807501

[B7] Arnoldini M. Vizcarra I. A. Peña-Miller R. Stocker N. Diard M. Vogel V.. (2014). Bistable expression of virulence genes in *Salmonella* leads to the formation of an antibiotic-tolerant subpopulation. PLoS. Biol. 12:e1001928. 10.1371/journal.pbio.1001928 25136970 PMC4138020

[B8] Bakardjiev A. I. Theriot J. A. Portnoy D. A. (2006). *Listeria monocytogenes* traffics from maternal organs to the placenta and back. PLoS. Pathog. 2:e66. 10.1371/journal.ppat.0020066 16846254 PMC1483233

[B9] Balestrino D. Hamon M. A. Dortet L. Nahori M. A. Pizarro-Cerda J. Alignani D.. (2010). Single-cell techniques using chromosomally tagged fluorescent bacteria to study *Listeria monocytogenes* infection processes. Appl. Environ. Microbiol. 76, 3625–3636. 10.1128/AEM.02612-09 20363781 PMC2876438

[B10] Bates D. Maechler M. Bolker B. Walker S. (2014). Lme4: Linear Mixed-Effects Models Using Eigen and S4. J. Stat. Softw. 67, 1–48. 10.18637/jss.v067.i01

[B11] Bishop D. K. Hinrichs D. J. (1987). Adoptive transfer of immunity to *Listeria monocytogenes*. The influence of *in vitro* stimulation on lymphocyte subset requirements. J. Immunol. 139, 2005–2009. 3114382

[B12] Bruno J. C. Jr. Freitag N. E. (2010). Constitutive activation of PrfA tilts the balance of *Listeria monocytogenes* fitness towards life within the host versus environmental survival. PLoS ONE 5:e15138. 10.1371/journal.pone.0015138 21151923 PMC2998416

[B13] Camejo A. Buchrieser C. Couve E. Carvalho F. Reis O. Ferreira P.. (2009). *In vivo* transcriptional profiling of *Listeria monocytogenes* and mutagenesis identify new virulence factors involved in infection. PLoS. Pathog. 5:e1000449. 10.1371/journal.ppat.1000449 19478867 PMC2679221

[B14] Chakraborty T. Leimeister-Wächter M. Domann E. Hartl M. Goebel W. Nichterlein T.. (1992). Coordinate regulation of virulence genes in *Listeria monocytogenes* requires the product of the *prfA* gene. J. Bacteriol. 174, 568–574. 10.1128/jb.174.2.568-574.1992 1729245 PMC205751

[B15] Chaturongakul S. Boor K. J. (2004). RsbT and RsbV contribute to sigmaB-dependent survival under environmental, energy, and intracellular stress conditions in *Listeria monocytogenes*. Appl. Environ. Microbiol. 70, 5349–5356. 10.1128/AEM.70.9.5349-5356.2004 15345420 PMC520851

[B16] Chaturongakul S. Raengpradub S. Wiedmann M. Boor K. J. (2008). Modulation of stress and virulence in *Listeria monocytogenes*. Trends Microbiol. 16, 388–396. 10.1016/j.tim.2008.05.006 18619843 PMC3400534

[B17] Crim S. M. Griffin P. M. Tauxe R. Marder E. P. Gilliss D. Cronquist A. B.. (2015). Preliminary incidence and trends of infection with pathogens transmitted commonly through food - Foodborne Diseases Active Surveillance Network, 10 U.S. sites, 2006-2014. MMWR. Morb. Mortal. Wkly. Rep. 64, 495–499. Available online at: https://www.cdc.gov/mmwr/preview/mmwrhtml/mm6418a4.htm 25974634 PMC4584825

[B18] Currie A. Farber J. M. Nadon C. Sharma D. Whitfield Y. Gaulin C.. (2015). Multi-province listeriosis outbreak linked to contaminated deli meat consumed primarily in institutional settings, Canada, 2008. Foodborne. Pathog. Dis. 12, 645–652. 10.1089/fpd.2015.1939 26258258

[B19] De las Heras A. Cain R. J. Bielecka M. K. Vázquez-Boland J. A. (2011). Regulation of *Listeria* virulence: PrfA master and commander. Curr. Opin. Microbiol. 14, 118–127. 10.1016/j.mib.2011.01.005 21388862

[B20] de Valk H. Jacquet C. Goulet V. Vaillant V. Perra A. Simon F.. (2005). Surveillance of *Listeria* infections in Europe. Euro. Surveill. 10, 251–255. Available online at: http://www.eurosurveillance.org/images/dynamic/EQ/v05n04/v05n04.pdf 16282642

[B21] Eldar A. Elowitz M. B. (2010). Functional roles for noise in genetic circuits. Nature 467, 167–173. 10.1038/nature09326 20829787 PMC4100692

[B22] Elowitz M. B. Levine A. J. Siggia E. D. Swain P. S. (2002). Stochastic gene expression in a single cell. Science 297, 1183–1186. 10.1126/science.1070919 12183631

[B23] Ferreira A. O'Byrne C. P. Boor K. J. (2001). Role of sigma(B) in heat, ethanol, acid, and oxidative stress resistance and during carbon starvation in *Listeria monocytogenes*. Appl. Environ. Microbiol. 67, 4454–4457. 10.1128/AEM.67.10.4454-4457.2001 11571142 PMC93189

[B24] Freitag N. E. Rong L. Portnoy D. A. (1993). Regulation of the *prfA* transcriptional activator of *Listeria monocytogenes:* multiple promoter elements contribute to intracellular growth and cell-to-cell spread. Infect. Immun. 61, 2537–2544. 8388865 10.1128/iai.61.6.2537-2544.1993 PMC280881

[B25] Gaillard J. L. Berche P. Frehel C. Gouin E. Cossart P. (1991). Entry of *L. monocytogenes* into cells is mediated by internalin, a repeat protein reminiscent of surface antigens from gram-positive cocci. Cell 65, 1127–1141. 10.1016/0092-8674(91)90009-N 1905979

[B26] George S. E. Nguyen T. Geiger T. Weidenmaier C. Lee J. C. Liese J.. (2015). Phenotypic heterogeneity and temporal expression of the capsular polysaccharide in *Staphylococcus aureus*. Mol. Microbiol. 98, 1073–1088. 10.1111/mmi.13174 26303846

[B27] Gottlieb S. L. Newbern E. C. Griffin P. M. Graves L. M. Hoekstra R. M. Baker N. L.. (2006). Multistate outbreak of Listeriosis linked to turkey deli meat and subsequent changes in US regulatory policy. Clin. Infect. Dis. 42, 29–36. 10.1086/498113 16323088

[B28] Guariglia-Oropeza V. Orsi R. H. Yu H. Boor K. J. Wiedmann M. Guldimann C. (2014). Regulatory network features in *Listeria monocytogenes* -changing the way we talk. Front. Cell Infect. Microbiol. 4:14. 10.3389/fcimb.2014.00014 24592357 PMC3924034

[B29] Hain T. Hossain H. Chatterjee S. S. Machata S. Volk U. Wagner S.. (2008). Temporal transcriptomic analysis of the *Listeria monocytogenes* EGD-e SigmaB regulon. BMC Microbiol. 8:20. 10.1186/1471-2180-8-20 18226246 PMC2248587

[B30] Heiman K. E. Garalde V. B. Gronostaj M. Jackson K. A. Beam S. Joseph L. (2016). Multistate outbreak of listeriosis caused by imported cheese and evidence of cross-contamination of other cheeses, USA, 2012. *Epidemiol*. Infect. 144, 2698–2708. 10.1017/S095026881500117X PMC6527316 26122394

[B31] Herbert K. C. Foster S. J. (2001). Starvation survival in *Listeria monocytogenes:* characterization of the response and the role of known and novel components. Microbiology 147, 2275–2284. 10.1099/00221287-147-8-2275 11496004

[B32] Hu Y. Oliver H. F. Raengpradub S. Palmer M. E. Orsi R. H. Wiedmann M.. (2007). Transcriptomic and phenotypic analyses suggest a network between the transcriptional regulators HrcA and sigmaB in *Listeria monocytogenes*. Appl. Environ. Microbiol. 73, 7981–7991. 10.1128/AEM.01281-07 17965207 PMC2168140

[B33] Ilari A. Ceci P. Ferrari D. Rossi G. L. Chiancone E. (2002). Iron incorporation into *Escherichia coli* Dps gives rise to a ferritin-like microcrystalline core. J. Biol. Chem. 277, 37619–37623. 10.1074/jbc.M206186200 12163499

[B34] Johansson J. Mandin P. Renzoni A. Chiaruttini C. Springer M. Cossart P. (2002). An RNA thermosensor controls expression of virulence genes in *Listeria monocytogenes*. Cell 110, 551–561. 10.1016/S0092-8674(02)00905-4 12230973

[B35] Kaern M. Elston T. C. Blake W. J. Collins J. J. (2005). Stochasticity in gene expression: from theories to phenotypes. Nat. Rev. Genet. 6, 451–464. 10.1038/nrg1615 15883588

[B36] Kazmierczak M. J. Mithoe S. C. Boor K. J. Wiedmann M. (2003). *Listeria monocytogenes* sigma B regulates stress response and virulence functions. J. Bacteriol. 185, 5722–5734. 10.1128/JB.185.19.5722-5734.2003 13129943 PMC193959

[B37] Kuznetsova A. Bruun Brockhoff P. Bojesen Christensen H. (2016). lmerTest: Tests in Linear Mixed Effects Models. Available online at: https://CRAN.R-project.org/package=lmerTest

[B38] Lauer P. Chow M. Y. Loessner M. J. Portnoy D. A. Calendar R. (2002). Construction, characterization, and use of two *Listeria monocytogenes* site-specific phage integration vectors. J. Bacteriol. 184, 4177–4186. 10.1128/JB.184.15.4177-4186.2002 12107135 PMC135211

[B39] Lobel L. Sigal N. Borovok I. Belitsky B. R. Sonenshein A. L. Herskovits A. A. (2015). The metabolic regulator CodY links *Listeria monocytogenes* metabolism to virulence by directly activating the virulence regulatory gene *prfA*. Mol. Microbiol. 95, 624–644. 10.1111/mmi.12890 25430920 PMC4329120

[B40] McIntyre L. Wilcott L. Naus M. (2015). Listeriosis outbreaks in British Columbia, Canada, caused by soft ripened cheese contaminated from environmental sources. Biomed. Res. Int. 2015:131623. 10.1155/2015/131623 25918702 PMC4396127

[B41] Mengaud J. Dramsi S. Gouin E. Vazquez-Boland J. A. Milon G. Cossart P. (1991). Pleiotropic control of *Listeria monocytogenes* virulence factors by a gene that is autoregulated. Mol. Microbiol. 5, 2273–2283. 10.1111/j.1365-2958.1991.tb02158.x 1662763

[B42] Milohanic E. Glaser P. Coppée J. Y. Frangeul L. Vega Y. Vazquez-Boland J. A.. (2003). Transcriptome analysis of *Listeria monocytogenes* identifies three groups of genes differently regulated by PrfA. Mol. Microbiol. 47, 1613–1625. 10.1046/j.1365-2958.2003.03413.x 12622816

[B43] Monk I. R. Gahan C. G. Hill C. (2008). Tools for functional postgenomic analysis of *Listeria monocytogenes*. Appl. Environ. Microbiol. 74, 3921–3934. 10.1128/AEM.00314-08 18441118 PMC2446514

[B44] Mujahid S. Orsi R. H. Vangay P. Boor K. J. Wiedmann M. (2013). Refinement of the *Listeria monocytogenes* sigmaB regulon through quantitative proteomic analysis. Microbiology 159, 1109–1119. 10.1099/mic.0.066001-0 23618998 PMC3709693

[B45] Nadon C. A. Bowen B. M. Wiedmann M. Boor K. J. (2002). Sigma B contributes to PrfA-mediated virulence in *Listeria monocytogenes*. Infect. Immun. 70, 3948–3952. 10.1128/IAI.70.7.3948-3952.2002 12065541 PMC128067

[B46] O'Byrne C. P. Karatzas K. A. (2008). The role of sigma B (sigma B) in the stress adaptations of *Listeria monocytogenes:* overlaps between stress adaptation and virulence. Adv. Appl. Microbiol. 65, 115–140. 10.1016/S0065-2164(08)00605-9 19026864

[B47] Olesen I. Thorsen L. Jespersen L. (2010). Relative transcription of *Listeria monocytogenes* virulence genes in liver pates with varying NaCl content. Int. J. Food. Microbiol. 141(Suppl. 1), S60–S68. 10.1016/j.ijfoodmicro.2010.01.042 20206397

[B48] Oliver H. F. Orsi R. H. Ponnala L. Keich U. Wang W. Sun Q.. (2009). Deep RNA sequencing of *L. monocytogenes* reveals overlapping and extensive stationary phase and sigma B-dependent transcriptomes, including multiple highly transcribed noncoding RNAs. BMC Genomics 10:641. 10.1186/1471-2164-10-641 20042087 PMC2813243

[B49] Oliver H. F. Orsi R. H. Wiedmann M. Boor K. J. (2010). *Listeria monocytogenes* sigma B has a small core regulon and a conserved role in virulence but makes differential contributions to stress tolerance across a diverse collection of strains. Appl. Environ. Microbiol. 76, 4216–4232. 10.1128/AEM.00031-10 20453120 PMC2897421

[B50] Ollinger J. Bowen B. Wiedmann M. Boor K. J. Bergholz T. M. (2009). *Listeria monocytogenes* sigmaB modulates PrfA-mediated virulence factor expression. Infect. Immun. 77, 2113–2124. 10.1128/IAI.01205-08 19255187 PMC2681731

[B51] Olsen K. N. Larsen M. H. Gahan C. G. Kallipolitis B. Wolf X. A. Rea R.. (2005). The Dps-like protein Fri of *Listeria monocytogenes* promotes stress tolerance and intracellular multiplication in macrophage-like cells. Microbiology 151, 925–933. 10.1099/mic.0.27552-0 15758237

[B52] Ondrusch N. Kreft J. (2011). Blue and red light modulates SigB-dependent gene transcription, swimming motility and invasiveness in *Listeria monocytogenes*. PLoS ONE 6:e16151. 10.1371/journal.pone.0016151 21264304 PMC3019169

[B53] Polidoro M. De B. D. Montagnini B. Guarrera L. Cavallo S. Valenti P.. (2002). The expression of the dodecameric ferritin in *Listeria* spp. is induced by iron limitation and stationary growth phase. Gene 296, 121–128. 10.1016/S0378-1119(02)00839-9 12383509

[B54] Popovic I. Heron B. Covacin C. (2014). Listeria: an Australian perspective (2001-2010). Foodborne. Pathog. Dis. 11, 425–432. 10.1089/fpd.2013.1697 24697613

[B55] Raengpradub S. Wiedmann M. Boor K. J. (2008). Comparative analysis of the sigma B-dependent stress responses in *Listeria monocytogenes* and *Listeria innocua* strains exposed to selected stress conditions. Appl. Environ. Microbiol. 74, 158–171. 10.1128/AEM.00951-07 18024685 PMC2223194

[B56] Raj A. van Oudenaarden A. (2008). Nature, nurture, or chance: stochastic gene expression and its consequences. Cell 135, 216–226. 10.1016/j.cell.2008.09.050 18957198 PMC3118044

[B57] Rauch M. Luo Q. Müller-Altrock S. Goebel W. (2005). SigB-dependent *in vitro* transcription of *prfA* and some newly identified genes of *Listeria monocytogenes* whose expression is affected by PrfA *in vivo*. J. Bacteriol. 187, 800–804. 10.1128/JB.187.2.800-804.2005 15629954 PMC543554

[B58] R Core Team (2015). R: A Language and Environment for Statistical Computing. Vienna: R Foundation for Statistical Computing.

[B59] Redfield A. R. Price C. W. (1996). General stress transcription factor sigmaB of *Bacillus subtilis* is a stable protein. J. Bacteriol. 178, 3668–3670. 10.1128/jb.178.12.3668-3670.1996 8655572 PMC178144

[B60] Reniere M. L. Whiteley A. T. Hamilton K. L. John S. M. Lauer P. Brennan R. G.. (2015). Glutathione activates virulence gene expression of an intracellular pathogen. Nature 517, 170–173. 10.1038/nature14029 25567281 PMC4305340

[B61] Ribeiro V. B. Mujahid S. Orsi R. H. Bergholz T. M. Wiedmann M. Boor K. J.. (2014). Contributions of sigma(B) and PrfA to *Listeria monocytogenes* salt stress under food relevant conditions. Int. J. Food. Microbiol. 177, 98–108. 10.1016/j.ijfoodmicro.2014.02.018 24631633 PMC8007333

[B62] Ringus D. L. Ivy R. A. Wiedmann M. Boor K. J. (2012). Salt stress-induced transcription of sigmaB- and CtsR-regulated genes in persistent and non-persistent *Listeria monocytogenes* strains from food processing plants. Foodborne. Pathog. Dis. 9, 198–206. 10.1089/fpd.2011.1000 22216988

[B63] Ripio M. T. Dominguez-Bernal G. Lara M. Suarez M. Vazquez-Boland J. A. (1997). A Gly145Ser substitution in the transcriptional activator PrfA causes constitutive overexpression of virulence factors in *Listeria monocytogenes*. J. Bacteriol. 179, 1533–1540. 10.1128/jb.179.5.1533-1540.1997 9045810 PMC178863

[B64] Schwab U. Bowen B. Nadon C. Wiedmann M. Boor K. J. (2005). The *Listeria monocytogenes prfA*P2 promoter is regulated by sigma B in a growth phase dependent manner. FEMS Microbiol. Lett. 245, 329–336. 10.1016/j.femsle.2005.03.025 15837390

[B65] Shetron-Rama L. M. Mueller K. Bravo J. M. Bouwer H. G. Way S. S. Freitag N. E. (2003). Isolation of *Listeria monocytogenes* mutants with high-level *in vitro* expression of host cytosol-induced gene products. Mol. Microbiol. 48, 1537–1551. 10.1046/j.1365-2958.2003.03534.x 12791137

[B66] Somolinos M. Espina L. Pagan R. Garcia D. (2010). *sigB* absence decreased *Listeria monocytogenes* EGD-e heat resistance but not its Pulsed Electric Fields resistance. Int. J. Food. Microbiol. 141, 32–38. 10.1016/j.ijfoodmicro.2010.04.023 20493572

[B67] Speer B. (2006). Pet chicken medicine and surgery, in NAVC Proceedings. Gainsville, FL.

[B68] Stewart M. K. Cookson B. T. (2014). Mutually repressing repressor functions and multi-layered cellular heterogeneity regulate the bistable *Salmonella fliC* census. Mol. Microbiol. 94, 1272–1284. 10.1111/mmi.12828 25315056 PMC4262692

[B69] Sue D. Boor K. J. Wiedmann M. (2003). Sigma(B)-dependent expression patterns of compatible solute transporter genes *opuCA* and *lmo1421* and the conjugated bile salt hydrolase gene *bsh* in *Listeria monocytogenes*. Microbiology 149, 3247–3256. 10.1099/mic.0.26526-0 14600237

[B70] Sue D. Fink D. Wiedmann M. Boor K. J. (2004). sigmaB-dependent gene induction and expression in *Listeria monocytogenes* during osmotic and acid stress conditions simulating the intestinal environment. Microbiology 150, 3843–3855. 10.1099/mic.0.27257-0 15528669

[B71] Tang S. Orsi R. H. den Bakker H. C. Wiedmann M. Boor K. J. Bergholz T. M. (2015). Transcriptomic analysis of the adaptation of *Listeria monocytogenes* to growth on vacuum-packed cold smoked salmon. Appl. Environ. Microbiol. 81, 6812–6824. 10.1128/AEM.01752-15 26209664 PMC4561693

[B72] Toledo-Arana A. Dussurget O. Nikitas G. Sesto N. Guet-Revillet H. Balestrino D.. (2009). The *Listeria* transcriptional landscape from saprophytism to virulence. Nature 459, 950–956. 10.1038/nature08080 19448609

[B73] Trost M. Wehmhöner D. Kärst U. Dieterich G. Wehland J. Jansch L. (2005). Comparative proteome analysis of secretory proteins from pathogenic and nonpathogenic *Listeria* species. Proteomics 5, 1544–1557. 10.1002/pmic.200401024 15838904

[B74] Utratna M. Cosgrave E. Baustian C. Ceredig R. O'Byrne C. (2012). Development and optimization of an EGFP-based reporter for measuring the general stress response in *Listeria monocytogenes*. Bioeng. Bugs 3, 93–103. 10.4161/bbug.19476 22539028 PMC3357339

[B75] Utratna M. Shaw I. Starr E. O'Byrne C. P. (2011). Rapid, transient, and proportional activation of sigma(B) in response to osmotic stress in *Listeria monocytogenes*. Appl. Environ. Microbiol. 77, 7841–7845. 10.1128/AEM.05732-11 21890665 PMC3209154

[B76] Warton D. I. Hui F. K. (2011). The arcsine is asinine: the analysis of proportions in ecology. Ecology 92, 3–10. 10.1890/10-0340.1 21560670

[B77] Wickham H. (2009). ggplot2: Elegant Graphics for Data Analysis. New York, NY: Springer.

[B78] Wickham H. (2011). The split-apply-combine strategy for data analysis. J. Stat. Softw. 40, 1–29. 10.18637/jss.v040.i01

[B79] Wiedmann M. Arvik T. J. Hurley R. J. Boor K. J. (1998). General stress transcription factor sigmaB and its role in acid tolerance and virulence of *Listeria monocytogenes*. J. Bacteriol. 180, 3650–3656. 9658010 10.1128/jb.180.14.3650-3656.1998 PMC107335

[B80] Wong K. K. Freitag N. E. (2004). A novel mutation within the central *Listeria monocytogenes* regulator PrfA that results in constitutive expression of virulence gene products. J. Bacteriol. 186, 6265–6276. 10.1128/JB.186.18.6265-6276.2004 15342597 PMC515134

