# Supplementary material for: Stochastic and Differential Activation of σB and PrfA in Listeria monocytogenes at the Single Cell Level under Different Environmental Stress Conditions
Source: Front Microbiol. 2017 Mar 14;8:348. doi: 10.3389/fmicb.2017.00348 (PMC5349113; doi:10.3389/fmicb.2017.00348)
Supplement: Supplementary file 2 [file Image1.pdf]

Supplementary file

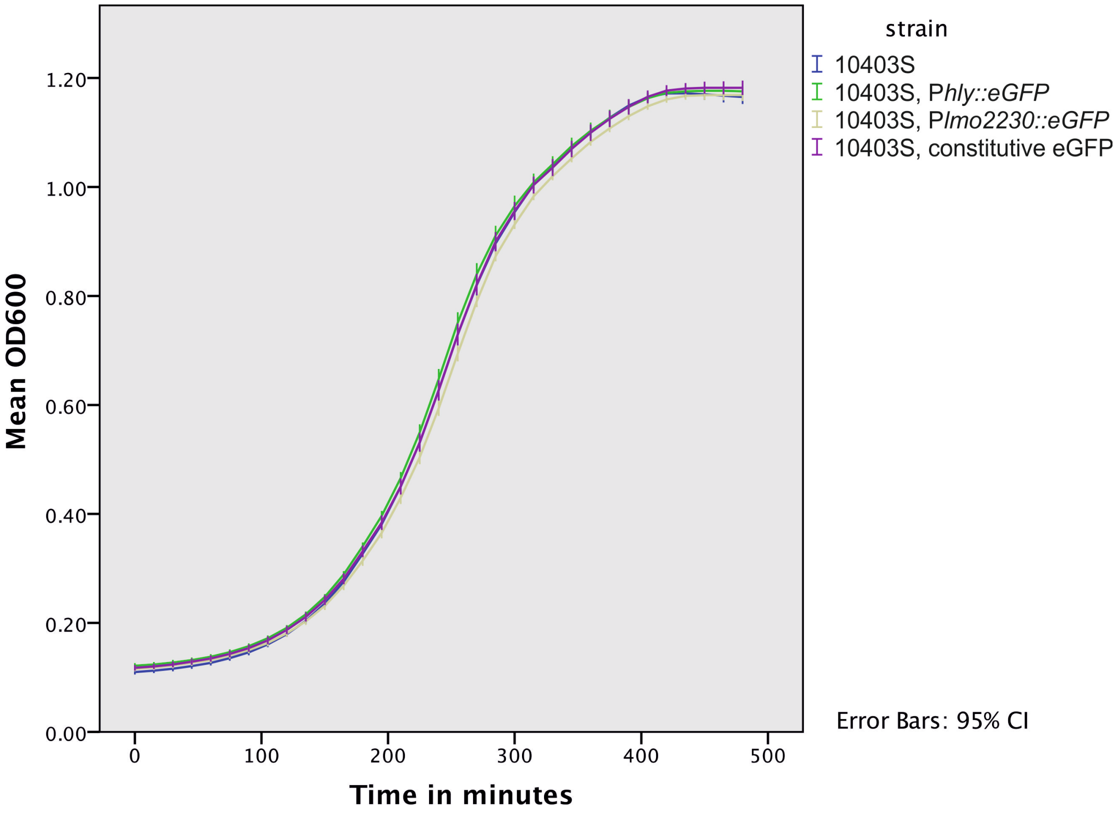

Supplementary Figure 1. Growth curves of *L. monocytogenes* 10403S, the PrfA reporter 10403S, P*hly::eGFP*, the σ^B reporter 10403S, P*lmo2230::eGFP* and the 10403S, constitutive eGFP expressing strain over 500 minutes. Log phase cultures (OD_{600} = 0.4) were diluted 1:40 into fresh BHI and the OD_{600} was measured every 15 minutes over eight hours. The data represent three biological replicates with two technical replicates each.

A Buchanan curve was fit to the data using the package "nlsMbio" in R. A Kruskal-Wallis rank sum test was used to compare the maximal growth rates, p = 0.1681.

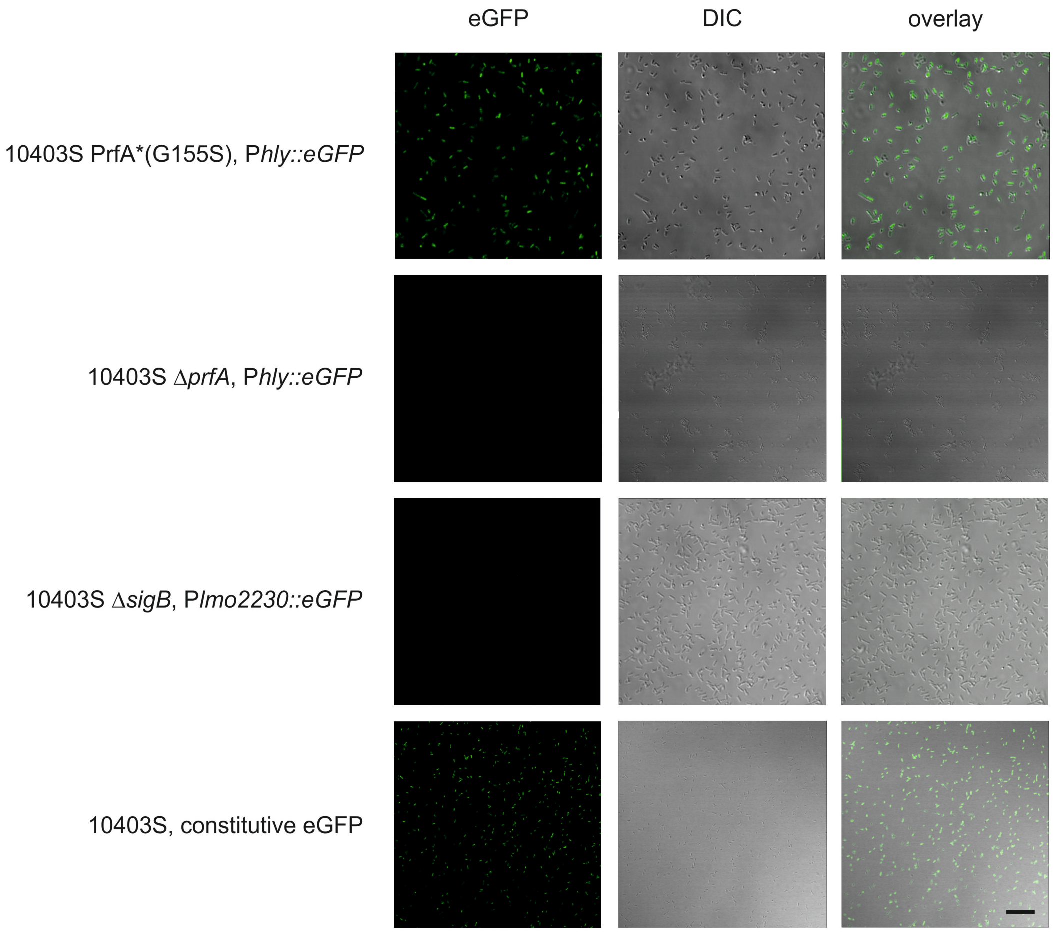

Supplementary Figure 2: representative confocal images of the control strains. The 10403S PrfA*(G155S), P*hly-eGFP* reporter and the 10403S, constitutive eGFP strains were grown to log phase (OD_{600}= 0.4) and imaged. The 10403S Δ*sigB*, P*lmo2230::eGFP* reporter was imaged after growing to stationary phase overnight. The log phase 10403S Δ*prfA*, P*hly::eGFP* reporter was exposed to heat stress in PBS as described in the materials and methods section and then imaged. Left column: eGFP fluorescence. Middle column: differential interference contrast (DIC). Right column: overlay of the eGFP fluorescence with the DIC image. Scale bar = 10 µm.

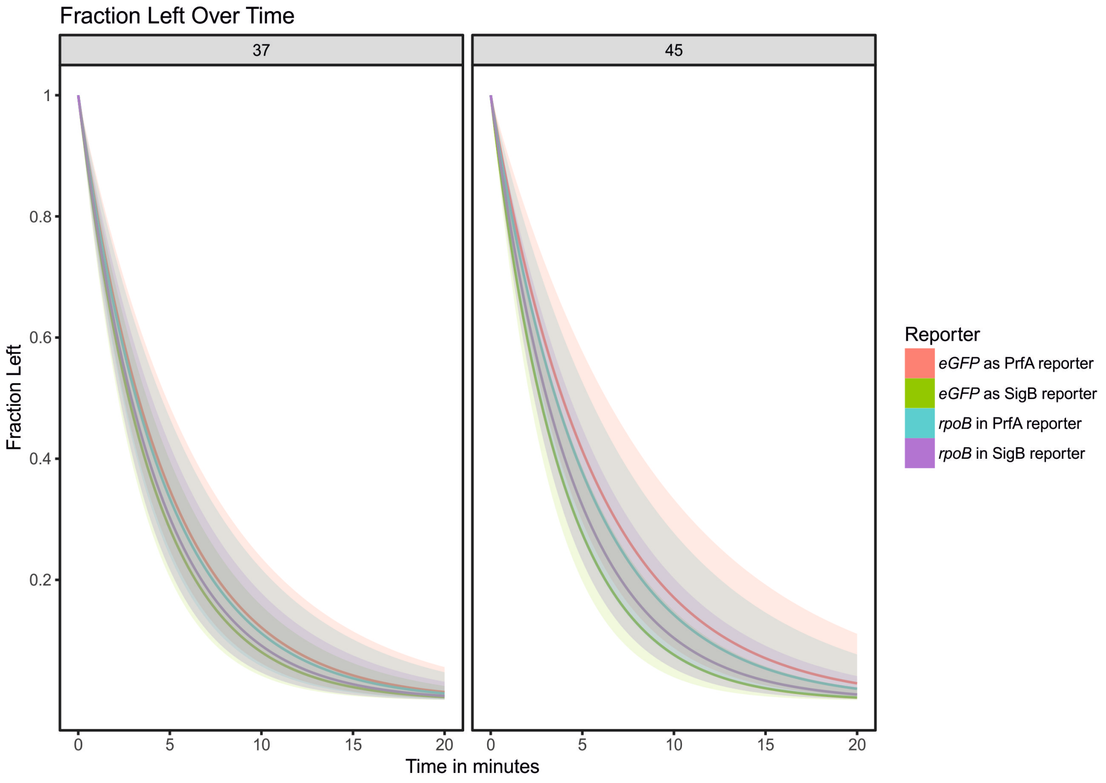

Supplementary Figure 3. mRNA stability of the reporter constructs. Transcription was stopped at t=0 through the addition of rifampicin (50 µg/ml) and the mRNA levels were determined by an RT-qPCR time series. There was no significant effect ($p=0.25$) of reporter (PrfA reporter or the σ^B reporter construct mRNA) on mRNA decay (as measured by the slope of the decay curve for the two reporters) using the data for both temperatures. The shading around the curves represents the 95% confidence interval.

Supplementary Table 1 | Primers used in this study

Name	Description	Sequence	Primer Pair Amplification Efficiency (Tm)[1]
CG20	P*lmo2230-eGFP* Utratna reverse	ATCGGTCGACTTATTTGTATAATTCATCCATTCCTAAAG	
CG22	P*lmo2230-eGFP* Utratna forward	ATGCGGATCCTTTCCGATATGTTTGTTTTTCCAGA	
CG17sBe	pPL2 MCS forward	GAAAGGGCCTCGTGATACGCCTA	
CG18sBe	pPL2 MCS reverse	GGTCGTTAAATAGCGACGTCAATACGACTC	
CG11sBe	P*hly* forward	ATGCGGATCCAAGTTACTTTTATGTGGAGGCATT	
CG12sBe	P*hly* reverse	GGGTTTCACTCTCCTTCTACATTT	
CG19	*eGFP* forward	AAATGTAGAAGGAGAGTGAAACCCATGGTTAGT AAAGGAGAGGAATTATTC	
CG20	*eGFP* reverse	ATCGGTCGACTTATTTGTATAATTCATCCATTCCTAAAG	
VGO-15-*sigB*-RT-F	qPCR	GCGCCGAATCAAAGAGTTAG	1.91 (55°C)
VGO-16-*sigB*-RT-R	qPCR	CCATCCGAATCAGCTTCAAT	
VGO-17-*lmo2230*-RT-F	qPCR	TGGGCGAAAAGACTTTCACT	1.86 (55°C)
VGO-18-*lmo2230*-RT-R	qPCR	GCTGGAAATTTTGGTGCAGT	
VGO-23-*rpoB*-RT-F	qPCR	TCGTCGTCTTCGTTCTGTTG	1.88 (55°C)
VGO-24-*rpoB*-RT-R	qPCR	GTTCGCCAAGTGGATTTGTT	
CG35*eGFP*qF	qPCR	GCGAAGGTGATGCTACTTACG	1.93 (60°C)
CG36*eGFP*qR	qPCR	CACGTATCCTTCTGGCATTG	
CG31*prfA*F	qPCR	TTGATACAGAAACATCGGTTGG	1.88 (55°C)
CG32*prfA*R	qPCR	AGCCAAGCTTCCCGTTAATC	
GC33*hly*F	qPCR	ATCTCAAGTGTGGCGTATGG	1.90 (55°C)
CG34*hly*R	qPCR	ACTTCATCTTTTGCGGAACC	

[1] Amplification efficiency of RT-qPCR primer pairs at the specified Tm
